# Supplementary material for: Seismic control of large prehistoric rockslides in the Eastern Alps
Source: Nat Commun. 2021 Feb 16;12:1059. doi: 10.1038/s41467-021-21327-9 (PMC7886888; doi:10.1038/s41467-021-21327-9)
Supplement: Supplementary file 1 — Supplementary Information [file 41467_2021_21327_MOESM1_ESM.pdf]

Supplementary Information

Title: Seismic control of large prehistoric rockslides in the Eastern Alps

Authors: Patrick Oswald[a]*, Michael Strasser[a], Christa Hammerl[b], Jasper Moernaut[a]

[a]University of Innsbruck, Department of Geology, 6020 Innsbruck, Austria

[b]Central Institute for Meteorology and Geodynamics, 1190 Vienna, Austria

corresponding author: Patrick Oswald, Patrick.Oswald@uibk.ac.at

Supplementary Figure 1: Seismicity map of the study area

Historically documented and measured earthquake epicentres since CE 1886[1] highlighting the concentration of seismic activity along a 25 km broad, W-E oriented belt in the central part of the study area. Lake Piburgersee depicts a more internal position and Lake Plansee a more external position in respect to the current seismic activity. The earthquakes have magnitudes up to M_L 5.3 (CE 1930 event; see also Supplementary Figure 6) and are concentrated at 5 to 10 km depths mainly at major thrust faults within the European plate[1]. Some of these faults are connected to Cretaceous-Tertiary faults exposed to the surface[2] featuring the potential of severe earthquakes. Major faults on the map are modified from a previous tectonic study[3]. Digital elevation model is derived from Land Tirol – data.tirol.gv.at.

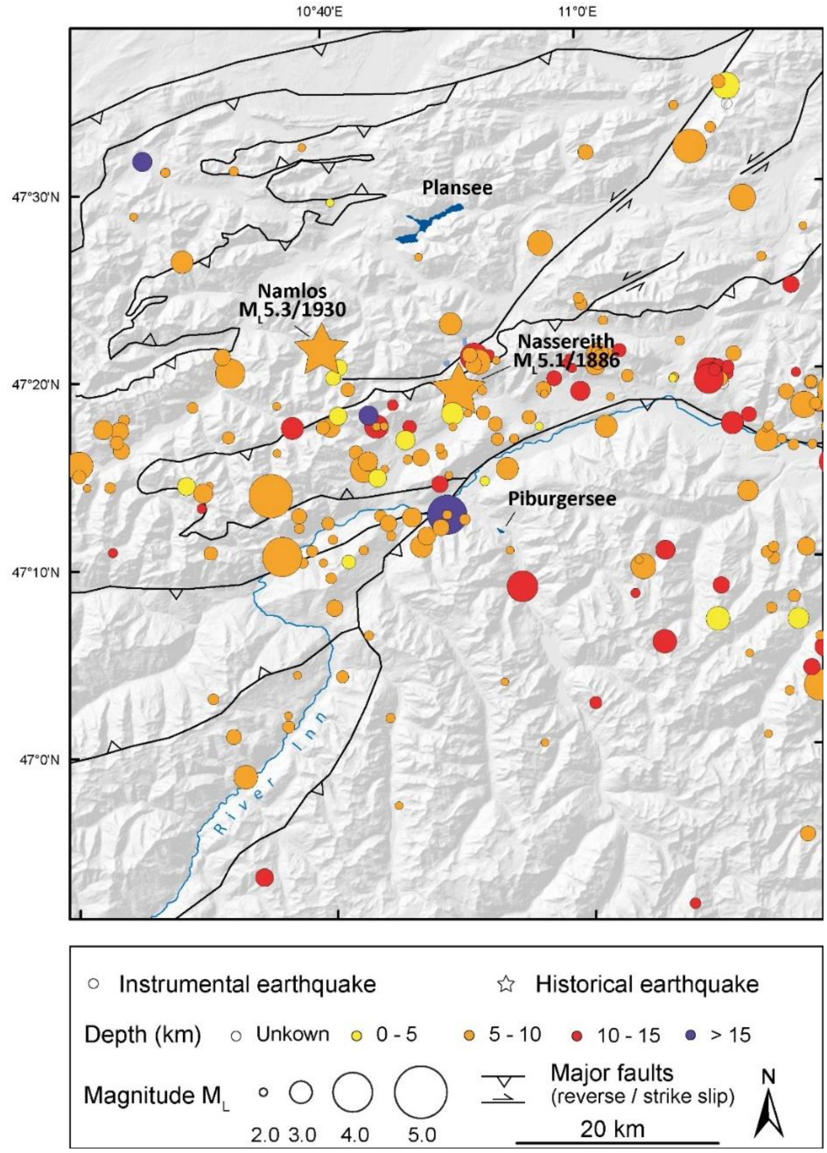

Supplementary Note 1

Lake Piburgersee

Lake Piburgersee (0.14 km²; 24 m depth) is located in the internal Eastern Alps within an area of recent seismic activity and surrounded by several rockslides (Supplementary Figure 1; Manuscript Figure 1a). The lake is situated within a glacially formed hanging valley at 913 m a.s.l. near the confluence of two main valleys (Inn valley and Ötz valley) and thus is not influenced by main inflowing rivers. The catchment is well defined to a 1.7 km² area with a single subaerial inflow in the West. Paleoseismic research on Piburgersee concentrates on the investigation of soft-sediment deformation structures (SSDS) within the sediment core taken in the deepest part of the lake. Reflection seismic data are not presented here as there is no seismic penetration of the 3.5kHz subbottom profiler signal due to free biogenic gas in the sediments derived from decomposition of abundant organic material[4].

Lake Plansee

Lake Plansee (4.24 km²; 68 m depth) is located more towards the Alpine foreland in the North, outside of the rockslide cluster and at the northern border of recent seismicity (Manuscript Figure 1a; Supplementary Figure 1). The lake lies within a glacially-overdeepened valley and forms the remnant of a larger pro-glacial lake during Late Glacial times[5]. The lake has been used as a reservoir for a local hydro power plant since CE 1908, inducing seasonal lake level changes. The lake is surrounded by an intensively jointed dolomite succession forming numerous talus slopes and alluvial fans, which subdivide the lake into five subbasins (see manuscript Figure 1). The focus of this paleoseismic study lies in the deepest central basin, where the entire sedimentary infill since the glacier's retreat is displayed in reflection seismic data (Supplementary Figure 7). The other subbasins are not considered in this study, because acoustic penetration is limited due to free biogenic gas in the sediments derived from decomposition of organic material entrained by river inflows. Moreover, sedimentation dynamics in these smaller subbasins are strongly affected by the proximity of several prograding alluvial fans on which debris flows repeatedly occur[6].

Supplementary Figure 2: Piburgersee core data

The sediment core of Piburgersee comprises several types of event deposits within the continuous glaciolacustrine-lacustrine sedimentary succession. The glaciolacustrine sediments are composed of light grey to brown, thick laminated to thin bedded, siliciclastic silts bearing normal graded sand intervals. The glaciolacustrine sediments are characterised by high magnetic susceptibility (MS) and Ca values, indicating detrital sedimentation. The element Ca is used due to its abundance in the feldspars of the surrounding paragneiss and due to its high count rates in XRF measurements compared with other elements e.g. Si. In addition, the Inc/Coh ratio, which can be used as a proxy for organic matter[7], is low supporting this interpretation. Lacustrine sedimentation is characterised by a dark brown, finely laminated to homogeneous mud, bearing organic colloids, chitin- and chert-rich remnants of microorganisms and macro remains of terrestrial plants. In core data, lacustrine sediments are characterised by low values in magnetic susceptibility and Ca, and high values in Inc/Coh. The different soft sediment deformation structures (SSDS; Supplementary Figure 5) and event deposits (Supplementary Figure 4) are indicated within the lacustrine organic-rich mud. Horizontal dashed, black lines between core image and CT image represent section breaks of the composite core. Ca and Inc/Coh data were processed by centre-log ratio transformation[8]. Inc/Coh ratio and partly Ca provide unreliable data within the rockfall-induced homogeneous turbidite (greyed-out wiggles) due to different core compaction or cracks related to coring disturbance of the different core sections. Core location is shown in Manuscript Figure 1b.

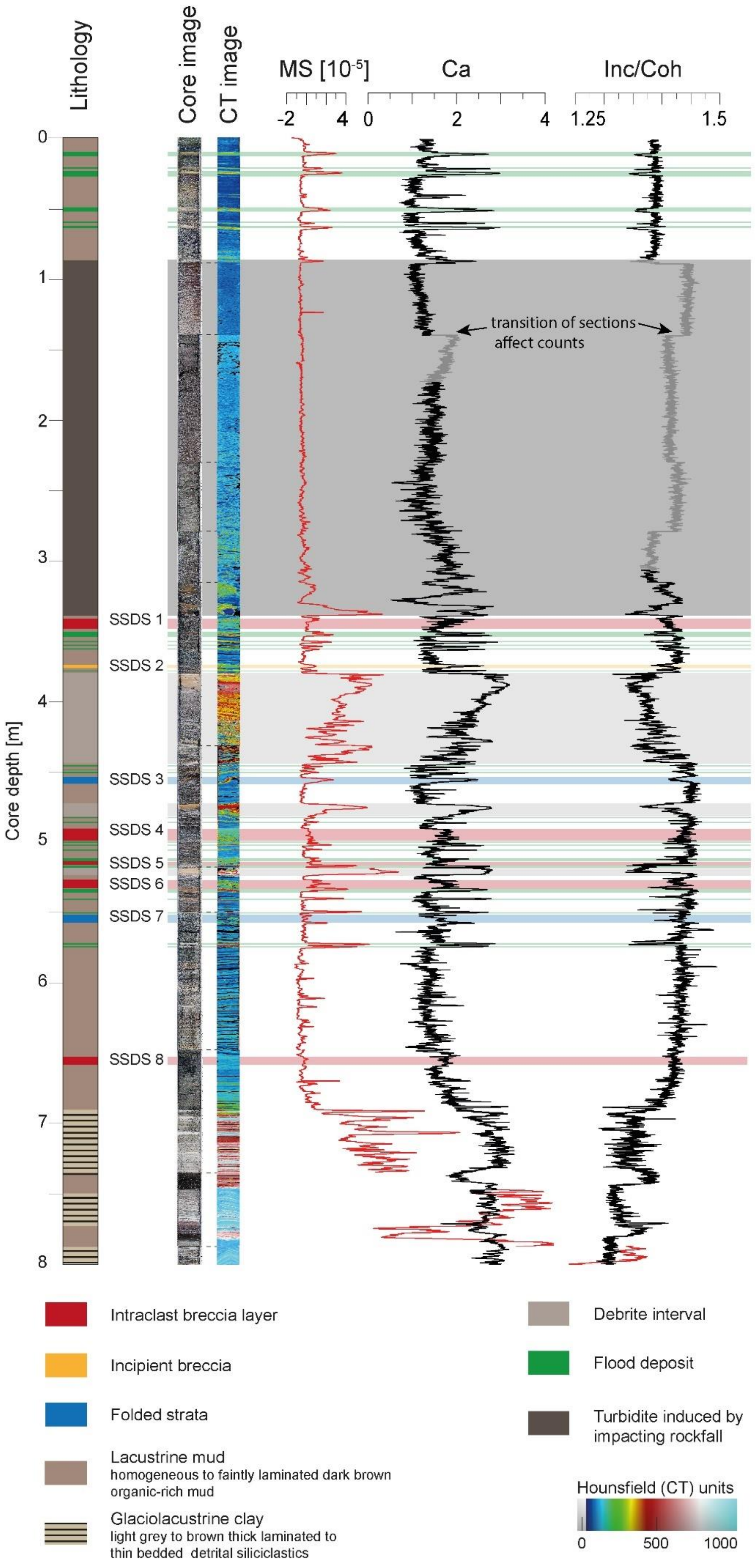

Lithology
Core image
CT image
MS [10-5]
Ca
Inc/Coh
-2
4
0
2
4
1.25
1.5
Core depth [m]
0
1
2
3
4
5
6
7
8
transition of sections affect counts
SSDS 1
SSDS 2
SSDS 3
SSDS 4
SSDS 5
SSDS 6
SSDS 7
SSDS 8
Intraclast breccia layer
Incipient breccia
Folded strata
Lacustrine mud
homogeneous to faintly laminated dark brown organic-rich mud
Glaciolacustrine clay
light grey to brown thick laminated to thin bedded detrital siliciclastics
Debrite interval
Flood deposit
Turbidite induced by impacting rockfall
Hounsfield (CT) units
0
500
1000

Supplementary Figure 3: Age-depth model of Piburgersee core

Age versus event-free depth plot of Piburgersee modelled with Bacon v2.4 software[9], combining 14 ^{14}C ages with xs^{210}Pb/^{137}Cs derived ages[4] (Supplementary Table 4). All event deposits > 0.5 cm are excluded. The respective event-free depths of soft sediment deformation structures (SSDS) are indicated by horizontal continuous lines. The sedimentation type change from glaciolacustrine to lacustrine sedimentation (horizontal dashed line at 294 cm) is set as boundary for the age-depth model facilitating the model to instantly shift sedimentation rates. Higher apparent sedimentation rates in the top 40 cm are explained by the poorly consolidated nature of the near-surface sediments. Lacustrine sedimentation has an overall mean event-free sedimentation rate of 0.025 cm/a, although sedimentation rates vary especially in the last 4.5 ka. The three plots above the age-depth model provide model input parameter and statistical information on the age-depth model quality[9].

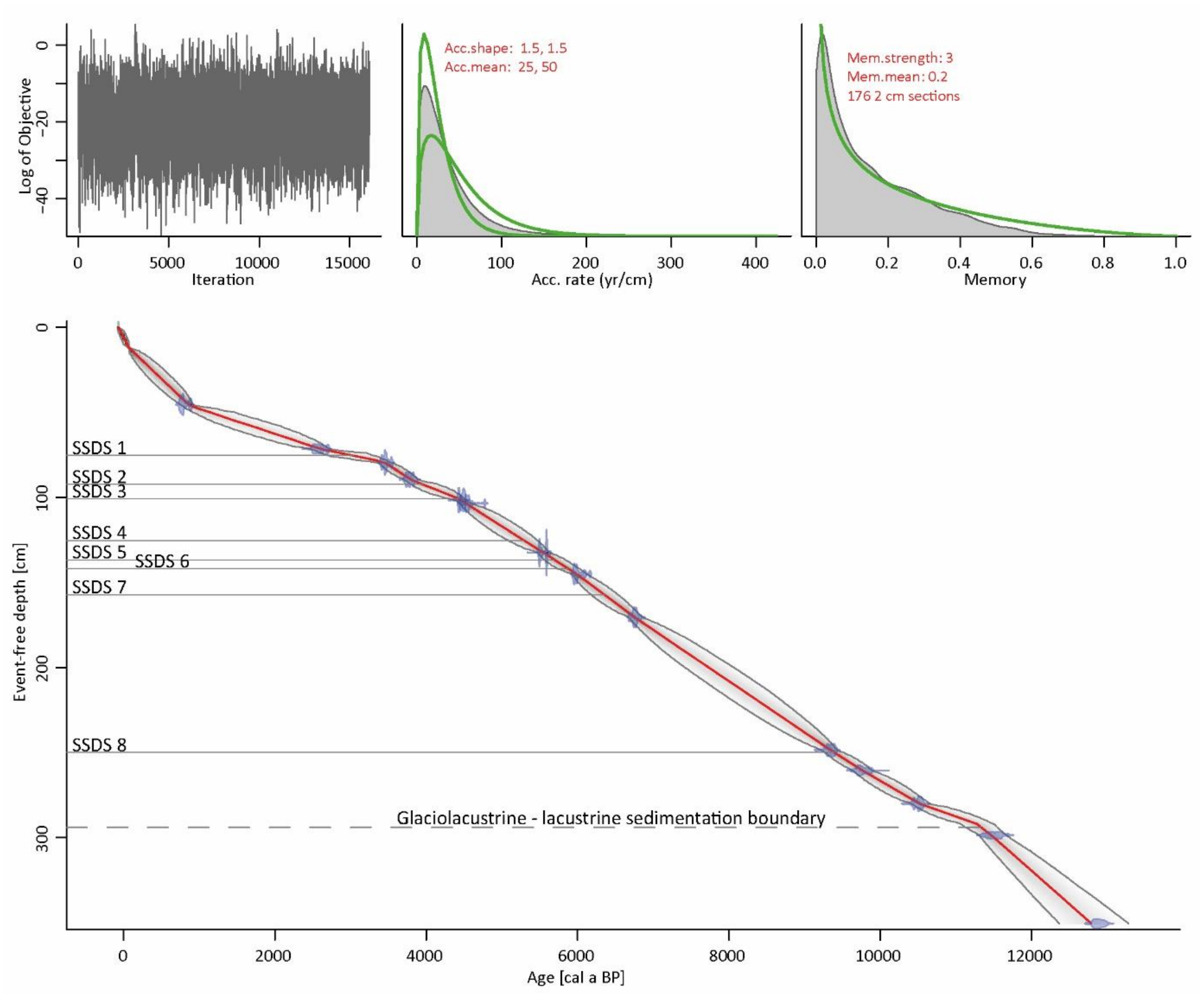

Supplementary Figure 4: Event deposits of Piburgersee not related to earthquakes

All event deposits were investigated to distinguish different types of instantaneous sedimentary processes affecting the lake system and to single out event beds related to earthquakes.

a) Surrounding area and geomorphological map of Piburgersee. The lake surroundings and the catchment mainly consist of Paragneiss rocks. Meta-granodiorites occur only in the Southeast due to the transported rock mass of the Habichen rockslide at the south-eastern side of the lake (d). The river inflow builds up a small delta (green) and depicts the origin of flood deposits (b). A 30 m wide subaerial failure scar south of the lake (red line) correspond to an impact depression and a bent compressional ridge occur at the basal slope attesting the failure of a local rock fall impacting the lake basin (c).

b) Flood deposits. Clastic, normal graded deposits with a coarse-grained base depict hyperpycnal flows released by high river discharge events (green bars). These flood deposits are characterised by peak values in magnetic susceptibility (MS) and Ca, both representative proxies for clastic input and further supported by troughs in the Inc/Coh ratio, here used as organic matter proxy[7]. Mineralogical composition derived from smear slides hints at remobilisation of Paragneiss rocks -containing quartz (qrz), feldspar (fsp), biotite (bio) and muscovite (mus)- outcropping in the lake catchment and along the small tributary (d). C/N ratios > 16 indicate a terrestrial origin of organic matter within recent flood deposits that were linked to historical high-discharge events in CE 1867, 1876 or 1877[4].

c) Turbidite induced by a local rock fall impacting the lake. A 2.5-meter thick mainly homogeneous deposit of reworked lake sediment is at 0.9 – 3.4 m sediment depth (Supplementary Figure 2). The turbidite base contains a deformed flood deposit and one gravel-sized Paragneiss clast indicating the involvement of an onshore gravitational mass movement remobilising a vast amount of lake sediment. The turbidite top is characterised by an extraordinary thick (0.5 cm) clay cap representing deposition of suspended fine-grained sediment in the aftermath of the event. The homogeneous character of the main middle part is indicated by constant petro-physical and geochemical core data (Supplementary Figure 2). XRF data shows variations at section breaks due to different degrees of compaction, water content or cracks in the core. These observations in combination with the age-depth model, bathymetric data and onshore elevation model (a) reveals that that a local rock fall at ~2.9 ka BP caused the extraordinary thick turbidite in the depocenter of the lake. The falling rock mass had a diameter of ~30 m based on the onshore elevation model and created a 3 m deep depression in front of a compressional ridge at the impact location (a).

d) Debrite interval potentially related to Habichen rockslide activity. The figure displays the thickest of three clastic intercalations present at 3.8 - 4.5 m, 4.7 - 4.8 m and 5.2 - 5.3 m sediment depth, whose appearance, texture, mineralogy, and thickness are different from typical flood deposits (Supplementary Figure 2). Each of these debrite intervals consist of multiple stacked high-density flow deposits representing onshore mass wasting surging into the lake basin. The displayed thickest debrite interval at ~4.4 ka BP purely consists of meta-granodiorite remnants containing feldspar (fsp) and amphibole (amp), which only occurs in the south-eastern catchment due to the Habichen rockslide (a). On top of this debrite interval, several mm-cm scaled debrites are stacked without any

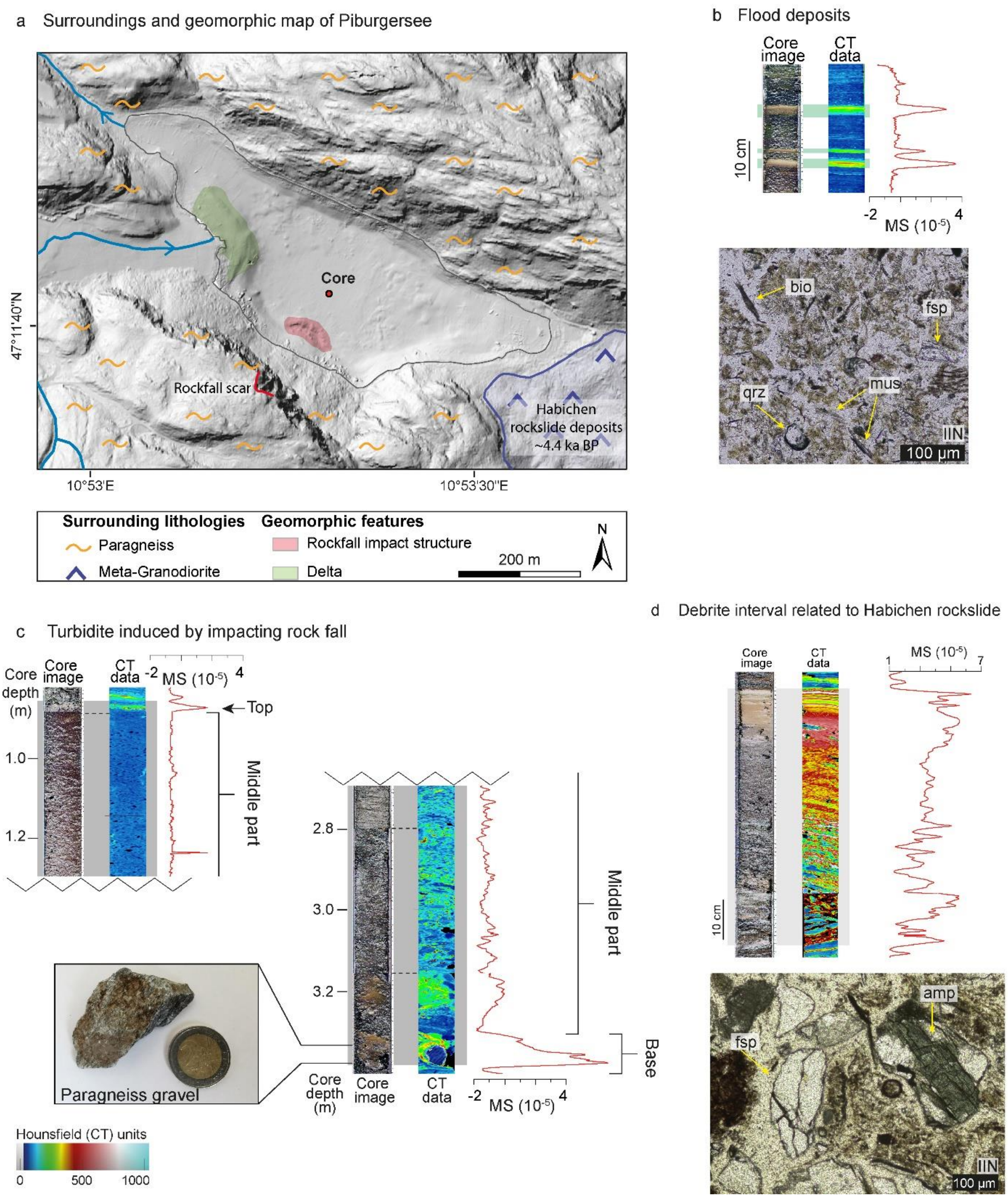

intercalation of lacustrine sediment hinting at a sudden availability of loose sediment in the aftermath of the main event, which got flushed into the lake during subsequent wet episodes. We interpret these observations that the debrite interval at ~4.4 ka BP relates to the emplacement of the Habichen rockslide. This age is in accordance with the estimated time frame of the Habichen rockslide activity (6.0 – 3.4 BP) based on backwater sediments in the main valley[10,11]. In contrast, the mineral assembly of the two smaller debrite intervals at ~6.1 and ~5.2 ka BP (Supplementary Figure 2) contains additional quartz and mica derived from Paragneiss rocks hinting at a mixed sediment source area. Thus, these small debrite intervals are interpreted as small-scale mass wasting in the distant south-eastern catchment composed of meta-granodiorite and partly remobilising also Paragneiss rocks from the primary near-lake catchment. Onshore digital elevation model is derived from Land Tirol – data.tirol.gv.at.

Supplementary Figure 5: Earthquake-induced soft sediment deformation structures (SSDS) in Piburgersee

A complete documentation of all eight SSDS occurring in the Holocene sedimentary succession of Piburgersee is displayed with core image (left) and CT data (right). Clast b-axis orientation of all intraclast breccias are displayed in rose diagrams in the lower figure part. Core location of the SSDS is found in Supplementary Figure 2. Event ages are shown in Supplementary Figure 2 and Supplementary Table 1. Different types of SSDS and the spectrum of increasing deformation from folded layer over incipient breccia to intraclast breccia are further explained in the main manuscript. Detailed description of the flood deposits, debrite interval and the turbidite induced by impacting rockfall is found in Supplementary Figure 4

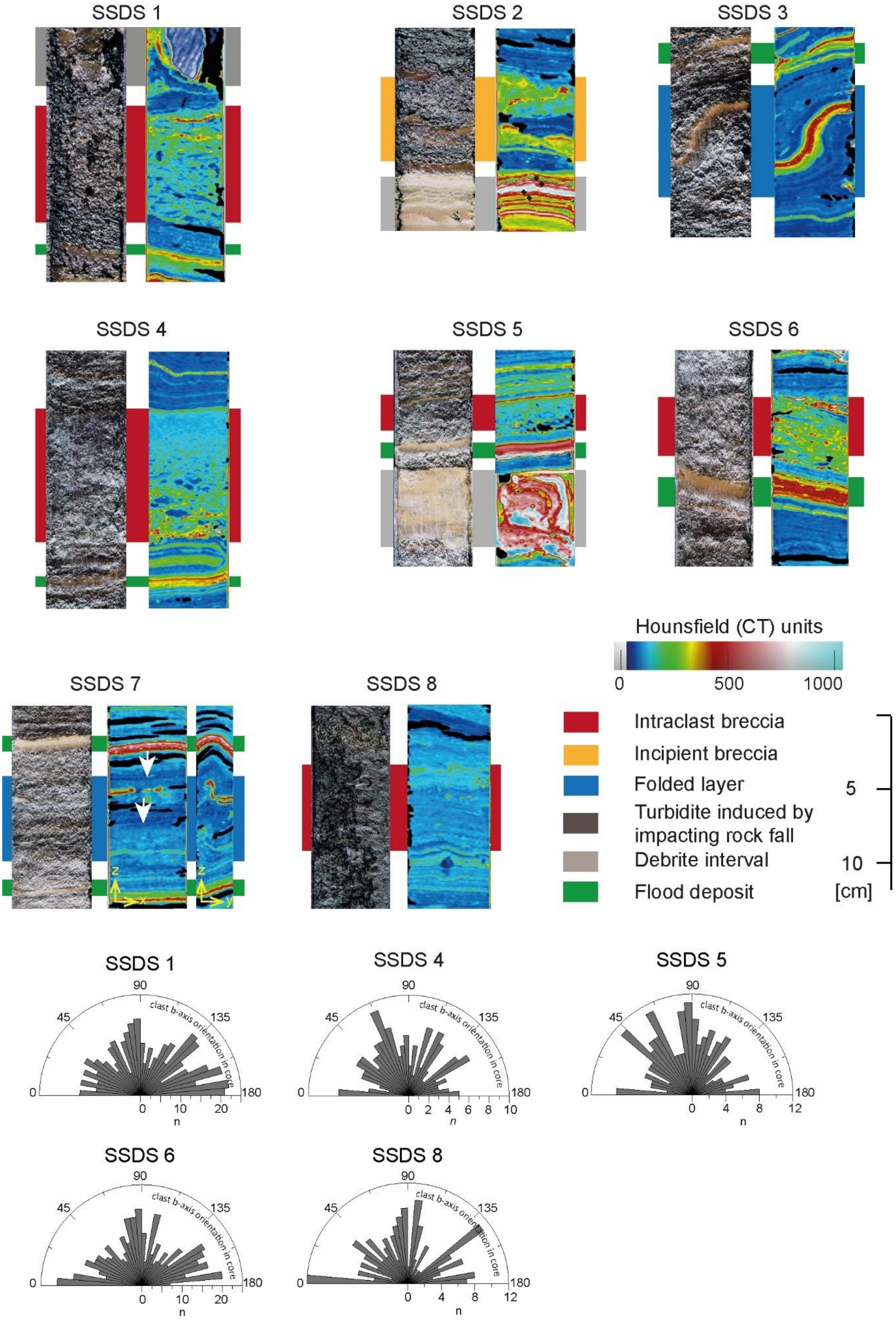
SSDS 1
SSDS 2
SSDS 3
SSDS 4
SSDS 5
SSDS 6
SSDS 7
SSDS 8
Hounsfield (CT) units
0
500
1000
Intraclast breccia
Incipient breccia
Folded layer
Turbidite induced by impacting rock fall
Debrite interval
Flood deposit
5
10
[cm]
clast b-axis orientation in core
0
45
90
135
180
n

Supplementary Discussion 1

In-situ soft-sediment deformation typically occurs at the sediment-water interface, because near-surface sediments have the lowest shear strength and are prone to deform by earthquake-induced shear stresses[12]. However, SSDS can also develop in deeper layers (dm to m scale) through excess pore pressure increase leading to liquefaction of susceptible (i.e. coarse-grained) layers[13]. In the case of Piburgersee, soft sediment deformation is interpreted to occur at the sediment-water interface and cogenetic development of intraclast breccia layers at multiple stratigraphic levels can be excluded based on the following sedimentological observations: i) normal grading of intraclast breccia layers indicating a gravitational settling process, ii) a thin clay cap on top of intraclast breccias representing the post-earthquake settlement of suspended fine sediment, iii) undeformed over- and underlying laminated lacustrine mud. Moreover, the documented SSDS in Piburgersee are attributed to in-situ deformation and do not originate from gravitational slope failure, because i) the b-clast orientations in intraclast breccias are random (Supplementary Figure 5) and gravitational flows would produce a dominant clast orientation[14], and ii) mean geochemical and petrophysical values of SSDS are similar to mean values of undeformed lacustrine mud and flood deposits in the core (Supplementary Figure 2); near-shore sediments are expected to yield a different overall composition and thus deposits of gravitational mass movements would exhibit different geochemical and petrophysical values. As folding ("slumping") can also occur at layers within a sedimentary sequence[13], determining the timing of SSDS2 and SSDS7 could be less confident. However, as SSDS 2 and SSDS 7 are overlain by undeformed, laminated lacustrine mud, in-situ folding at the sediment-water interface is the most plausible explanation.

In case of frequent earthquake occurrence and low sedimentation rate, it is possible that an earthquake-related SSDS can be overprinted by a subsequent earthquake[15]. This would generate a relatively thicker overall SSDS and only the most recent paleo-earthquake would be inferred. As the documented SSDS in Piburgersee have a comparable thickness, we do not expect overprinting to be a relevant process. In any case, if overprinting would have taken place, this would imply that the inferred period of enhanced paleo-seismicity (i.e. short recurrence intervals) could even have experienced more earthquakes than documented in our record.

The incipient breccia of SSDS 2 is characterised by a several cm thick shear zone with isoclinally folded and sheared flood deposits and lacustrine mud overlying a debrite interval containing micro faults with mm-scale normal displacements (Supplementary Figure 5; Manuscript Figure 2d;). Such brittle deformation requires dynamic loading e.g. by severe seismic shaking, as other potential causes such as rapid sediment loading can be excluded due to absence of thick instantaneous deposits above. The cogenetic, seismically-triggered development of liquefaction and brittle

deformation in the underlying unit can be explained by the contrasting physical properties of the sediment sequence before deformation, i.e soft lacustrine muds overlying a more competent debrite.

Supplementary Table 1: Piburgersee earthquake events

Earthquake ID	SSDS	Core depth [cm]	Event corrected depth [cm]	Modelled event ages [cal a BP]				SSDS type	Qualitative earthquake intensity
				mean	from	to	%		
EQ-1	1	344	75.5	3039	2716	3306	95	intraclast breccia	high
EQ-2	2	376.5	95.5	4133	3927	4327	95	incipient breccia	intermediate
EQ-3	3	458	108	4681	4528	4871	95	folded layer	low
EQ-4	4	493.5	132.5	5550	5474	5636	95	intraclast breccia	high
EQ-5	5	517	148.5	6092	5987	6252	95	intraclast breccia	high
EQ-6	6	530.5	154	6258	6093	6445	95	intraclast breccia	high
EQ-7	7	554.5	172.5	6823	6716	6964	95	folded layer	low
EQ-8	8	654.5	265	9939	9767	10133	95	intraclast breccia	high

Supplementary Figure 6: Calibration of the lakes' individual earthquake-recording threshold with historical earthquakes

Isoseismal maps of the two strongest, historical earthquakes M_L 5.3 Namlos in CE 1930 (left) and M_L 5.1 Nassereith in CE 1886 (right). We calibrated the intensity threshold required to leave specific sedimentary imprints in the studied lakes by the comparison of seismic intensities at the lake sites of these two earthquakes to the corresponding stratigraphic levels in the lake sedimentary sequence. Due to the lack of historical earthquake information in the direct vicinity of the lakes, the seismic intensities at the lake sites were interpolated from intensity data points (IDPs) of nearby villages. IDPs were obtained for the CE 1886 Nassereith earthquake (Supplementary Table 6) and the CE 1930 Namlos earthquake (Supplementary Data 1) by regional investigation of contemporary sources of villages nearby the lakes and assessment of the local intensities at each village based on the EMS-98[16]. Isoseismal maps were generated by interpolating between the IDPs using the inverse distance weighting (IDW) method in Esri ArcGIS and were used to estimate the seismic intensity at the studied lake sites. Onshore digital elevation model is derived from Land Tirol – data.tirol.gv.at.

The CE 1886 Nassereith earthquake produced a seismic intensity of V-V½ at both lakes and left no specific sedimentary imprints in those. In contrast, the CE 1930 Namlos earthquake, which also caused ground cracks near the epicentre and small-scale subaerial mass movements[17], reached a

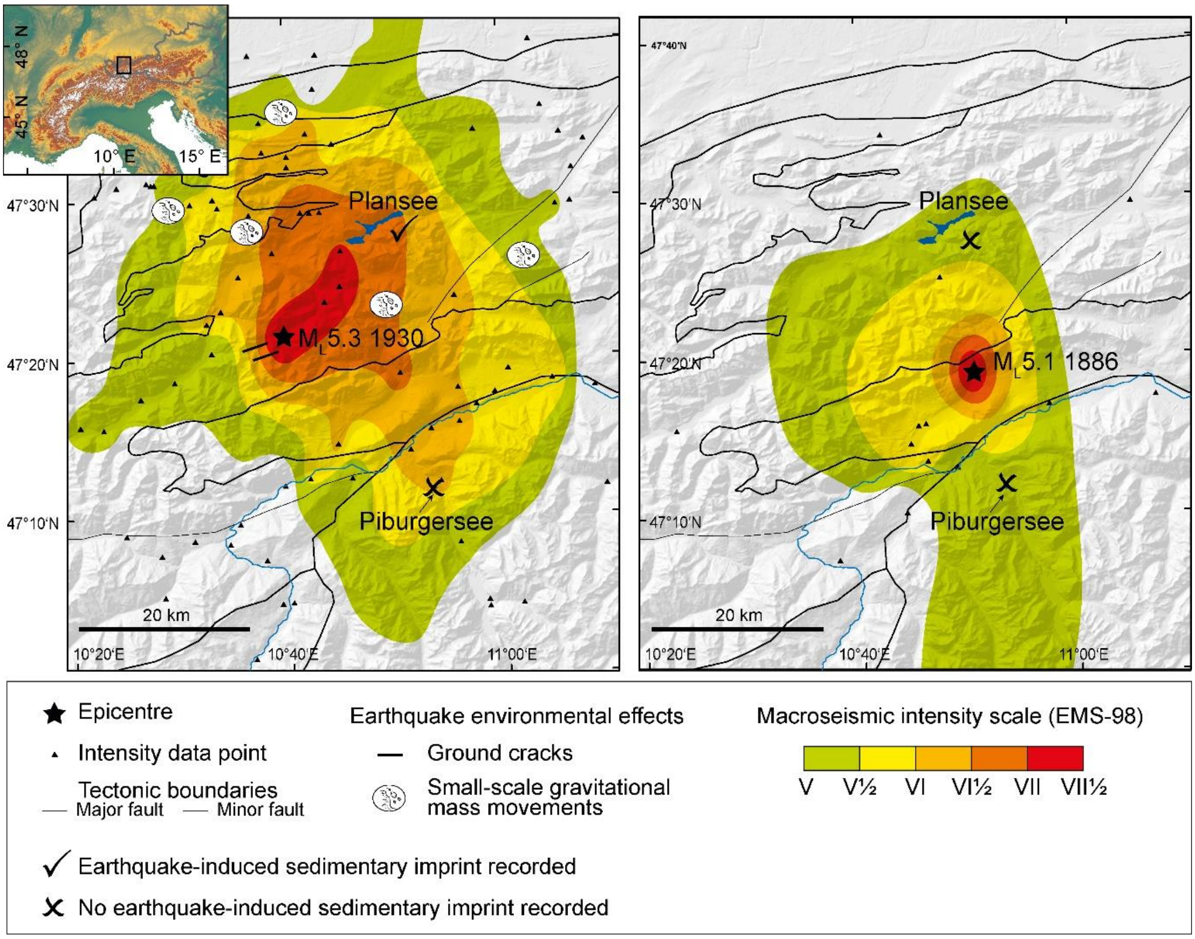

seismic intensity of VI½ – VII at Plansee where it produced multiple subaquatic mass-transport deposits (Supplementary Figure 9) and a cogenetic relatively thin amalgamated turbidite. It reached intensity VI at Piburgersee where no sedimentary imprint was left. Thus, for Plansee, the seismic intensity threshold for recording earthquakes by multiple MTDs is ~VI, which is comparable to previous studies in glacigenic lake basins [18–20]. For Piburgersee, the available data indicates that only earthquakes exceeding seismic intensity VI are capable of generating specific imprints in the water- and organic-rich mud.

Interpolation of IDPs of historical earthquakes is the most straightforward strategy to assess the earthquake-recording threshold intensity at the lake sites[21], when no instrumentally-recorded ground motion parameters are available, such as peak ground acceleration (PGA). Previous studies on SSDS have illustrated the advantage of using the quantitative PGA values over semi-quantitative intensity values, as PGA values may correlate with the deformation degree of SSDS [12,22]. However, in our study, the use of PGA would introduce extra uncertainties to our threshold calibrations and magnitude estimations, because i) PGA-intensity relations have large uncertainties in the upper magnitude range as they are derived from smaller earthquakes in the study area and ii) magnitude calculations of historical earthquakes are based on IDPs and therefore the workaround would be going from IDPs to earthquake magnitude and depth and then via a ground motion prediction equation (derived from smaller earthquakes) to a PGA value at the lake site. Thus, we consider the use of PGA in calibrating an earthquake-recording threshold for a lacustrine archive to be only appropriate when recent severe earthquakes are instrumentally recorded and adequate ground motion prediction equations exists for such relatively high magnitude events. However, future research in lacustrine paleoseismology should aim at implementing more quantitatively constrained ground motion parameters of prehistoric earthquakes.

Supplementary Figure 7: Mass-transport deposits (MTDs) in reflection seismic data of Plansee

Seismic profile of the central basin in Plansee showing multiple coeval mass-transport deposits (MTDs) and their corresponding event horizons A to K. MTDs were identified in reflection seismic data by their chaotic- to transparent seismic facies within the continuous, parallel reflections of undisturbed sediments[23]. A seismic-stratigraphic horizon was defined at the top of the pinch-out point of each MTD and mapped throughout the whole central basin. As these event horizons represent chronostratigraphic horizons, multiple MTDs in a single event horizon are considered as coeval. In Plansee, multiple coeval MTDs, a potential proxy for earthquakes[24], correspond to eleven event horizons (A to K; Supplementary Figure 9). Turbidites within theoretical vertical seismic resolution (~10 cm) occurring at event horizon C and E are characterised by a transparent facies and a ponding geometry (transparent coloured). The sediment core of Manuscript Figure 3 (black square) is projected into the seismic profile based on the core-to-seismic correlation (Supplementary Figure 10). The acoustic basement (dashed grey line) is interpreted as the approximate

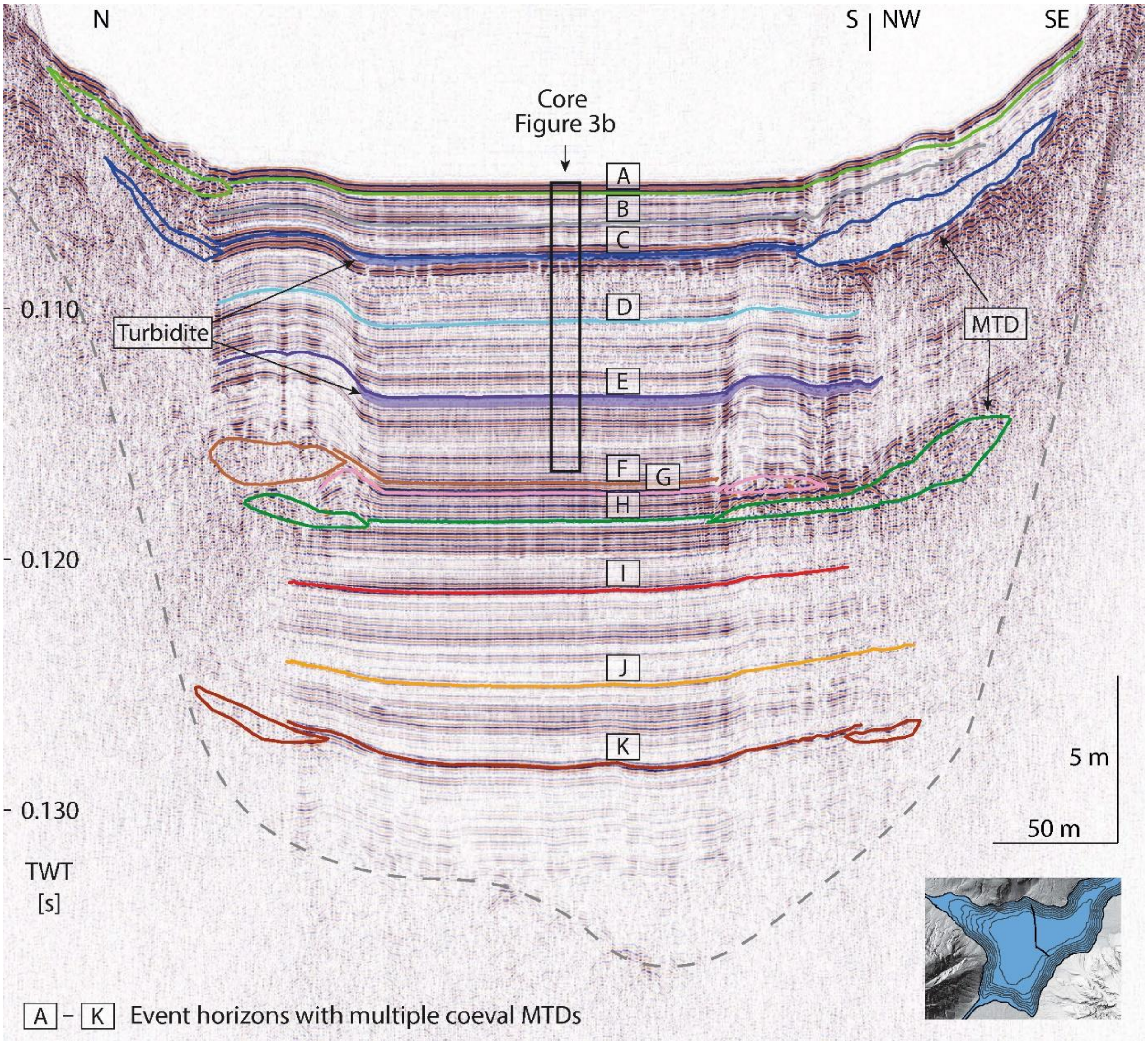

glacial erosional surface of the Last Glacial Maximum. Inset in the lower right corner shows the location of the seismic profile.

Supplementary Figure 8: Mass-transport deposit (MTD) types in reflection seismic data of Plansee

Two MTD types were identified based on seismic facies and amplitude characteristics. a) MTD type 1 is characterised by a chaotic facies with low to intermediate reflection amplitudes. Its top often has an irregular shape and its base locally truncates stratigraphically-deeper reflectors. b) MTD type 2 shows parallel to chaotic reflections with high amplitudes in the deposit. We interpret MTD type 1 as predominantly consisting of remobilised hemipelagic sediments and MTD type 2 of deltaic slope sediments. This interpretation is based on the MTD location in respect to its slope type and the MTD seismic facies, where a high reflection amplitude indicates increased sand content in the lacustrine mud. Deltaic slope failures can occur spontaneously without a clear external trigger[25]. However, when deltaic slope failures occur coevally on multiple slopes, an earthquake origin is considered the most likely trigger, as was documented for clastic-influenced lakes with high sedimentation rates[26].

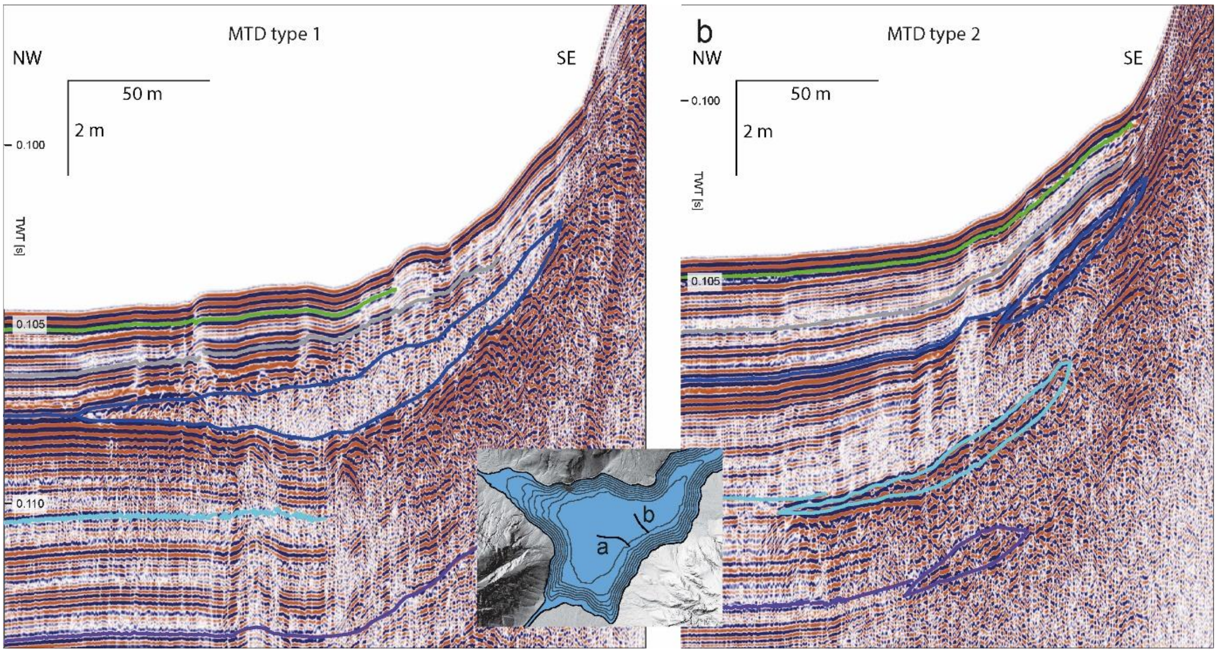

Supplementary Figure 9: Mass-transport deposit (MTD) distribution and thickness maps of event horizons in Plansee

Distribution- and thickness maps of all MTDs occurring in eleven event horizons within the reflection seismic data of Plansee. The extent and thickness of MTDs can be considered as approximations due to uncertainties related to MTD mapping given by the two-dimensional survey grid density, poor disentangling of MTD stacks and variable seismic penetration. MTD volumes are interpolated with a simple kriging technique and calculated based on the assumption of 1,500 m/s acoustic velocity. Each of the event horizons (A to K) contains 2 to 12 MTDs with total remobilised volumes up to 100,000 m³. Additional six event horizons (F to K) with multiple MTDs occur within the glaciolacustrine clays deposited before 10 ka BP, potentially indicating earthquakes during Late Glacial times and the early Holocene. Onshore digital elevation model is derived from Land Tirol – data.tirol.gv.at

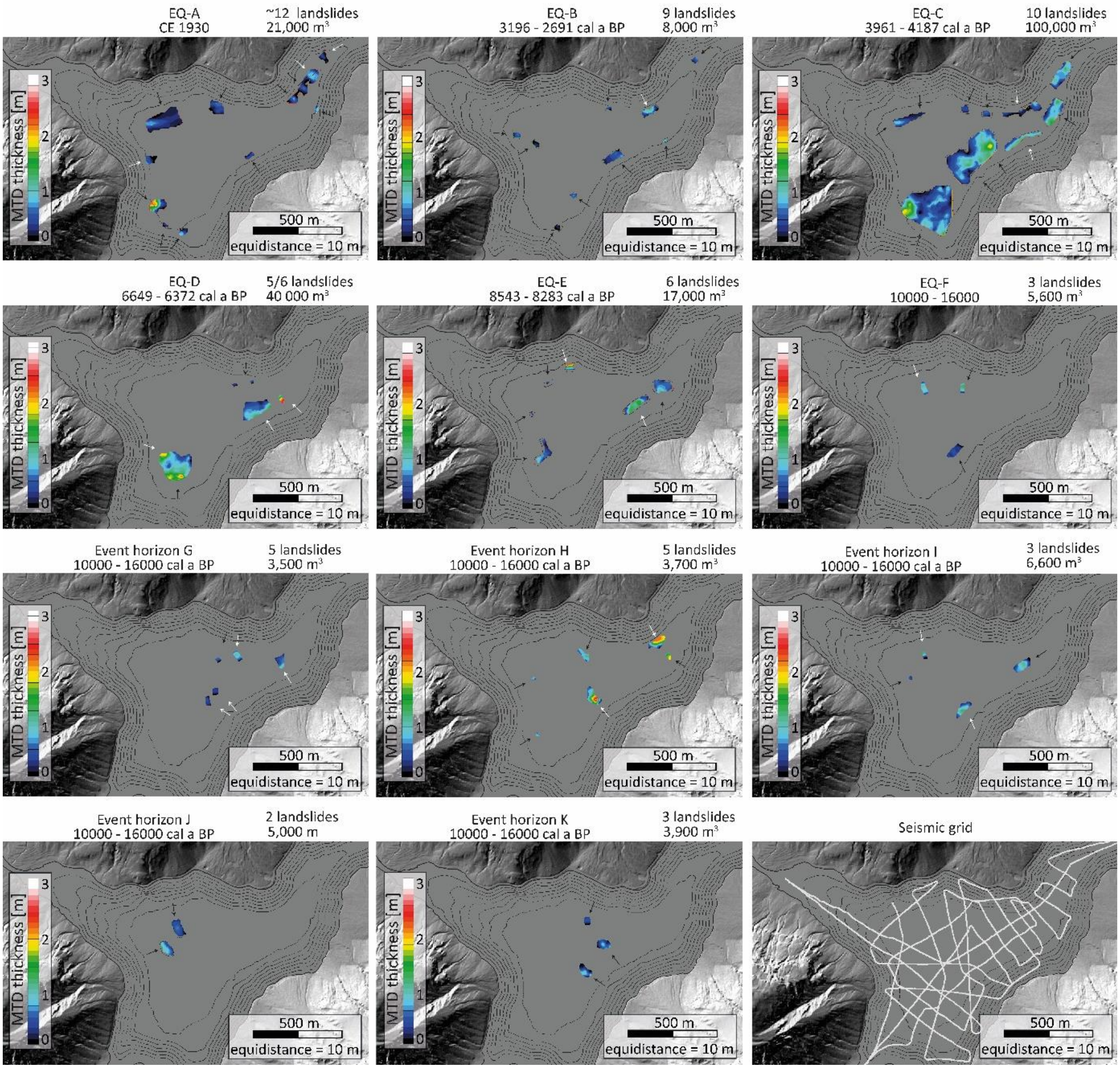

Supplementary Figure 10: Plansee core data and core-to-seismic correlation

The sediment core of Plansee contains several levels of amalgamated turbidites (red) and subsequent enhanced clastic input (grey) interpreted as postseismic landscape response within the continuous glaciolacustrine-lacustrine sedimentary succession. Glaciolacustrine clays are composed of light grey to brown, thick laminated to thin bedded, detrital carbonates and characterised by high lightness values (indicating brighter colours) and high bulk densities on average. Around 6.8 m core depth (~10 ka BP; Supplementary Figure 11) sedimentation changed to pure lacustrine sedimentation characterised by grey to ochre, finely laminated clayey silts with abundant detrital carbonates, diatoms and organic matter. The lacustrine clayey silts are frequently intercalated by flood or debris flow deposits mostly < 2 cm thick. A core-to-seismic correlation was conducted based on core bulk density and reflection patterns in seismics (bold black lines). Thick event deposits e.g. an amalgamated turbidite often have changes in bulk density, which represent good marker horizons to correlate with the reflection patterns in the seismic data. Horizontal dashed, black lines between core image and CT image represent section breaks of the composite core. Core location is shown in Manuscript Figure 1b.

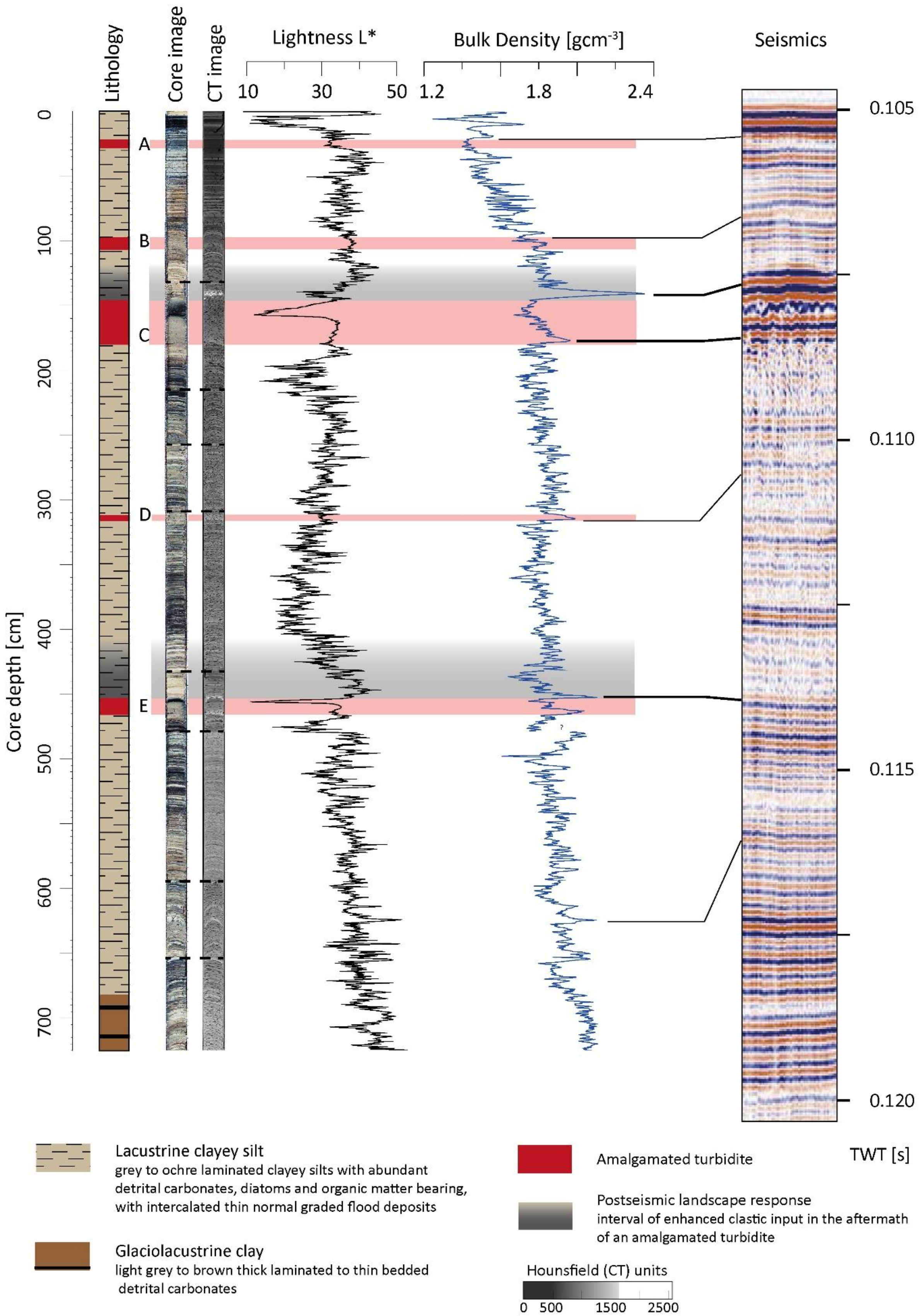
Lithology
Core image
CT image
Lightness L*
10 30 50
Bulk Density [gcm-3]
1.2 1.8 2.4
Seismics
Core depth [cm]
0
100
200
300
400
500
600
700
A
B
C
D
E
0.105
0.110
0.115
0.120
TWT [s]
Lacustrine clayey silt
grey to ochre laminated clayey silts with abundant detrital carbonates, diatoms and organic matter bearing, with intercalated thin normal graded flood deposits
Glaciolacustrine clay
light grey to brown thick laminated to thin bedded detrital carbonates
Amalgamated turbidite
Postseismic landscape response
interval of enhanced clastic input in the aftermath of an amalgamated turbidite
Hounsfield (CT) units
0 500 1500 2500

278

Supplementary Figure 11: Age-depth model of Plansee core

Event-free age-depth model of Plansee using Bacon v2.4 software[9], combining 13 ^{14}C ages with $xs^{210}Pb/^{137}Cs$ derived ages (Supplementary Figure 13; Supplementary Table 5). All event deposits > 0.5 cm are excluded. The sedimentation type change from glaciolacustrine to lacustrine sedimentation (horizontal dashed line at 511 cm) is set as boundary for the age-depth model facilitating the model to instantly shift sedimentation rates. Lacustrine sedimentation has an overall mean event-free sedimentation rate of 0.045 cm/a. Higher apparent sedimentation rates in the top 40 cm are explained by the poorly consolidated nature and high water content of the near-surface sediments and by the influence of artificial lake level changes since CE 1902 enhancing erosion and remobilisation of coastal sediment. The three plots above the age-depth model provide model input parameter and statistical information on the age-depth model quality[9].

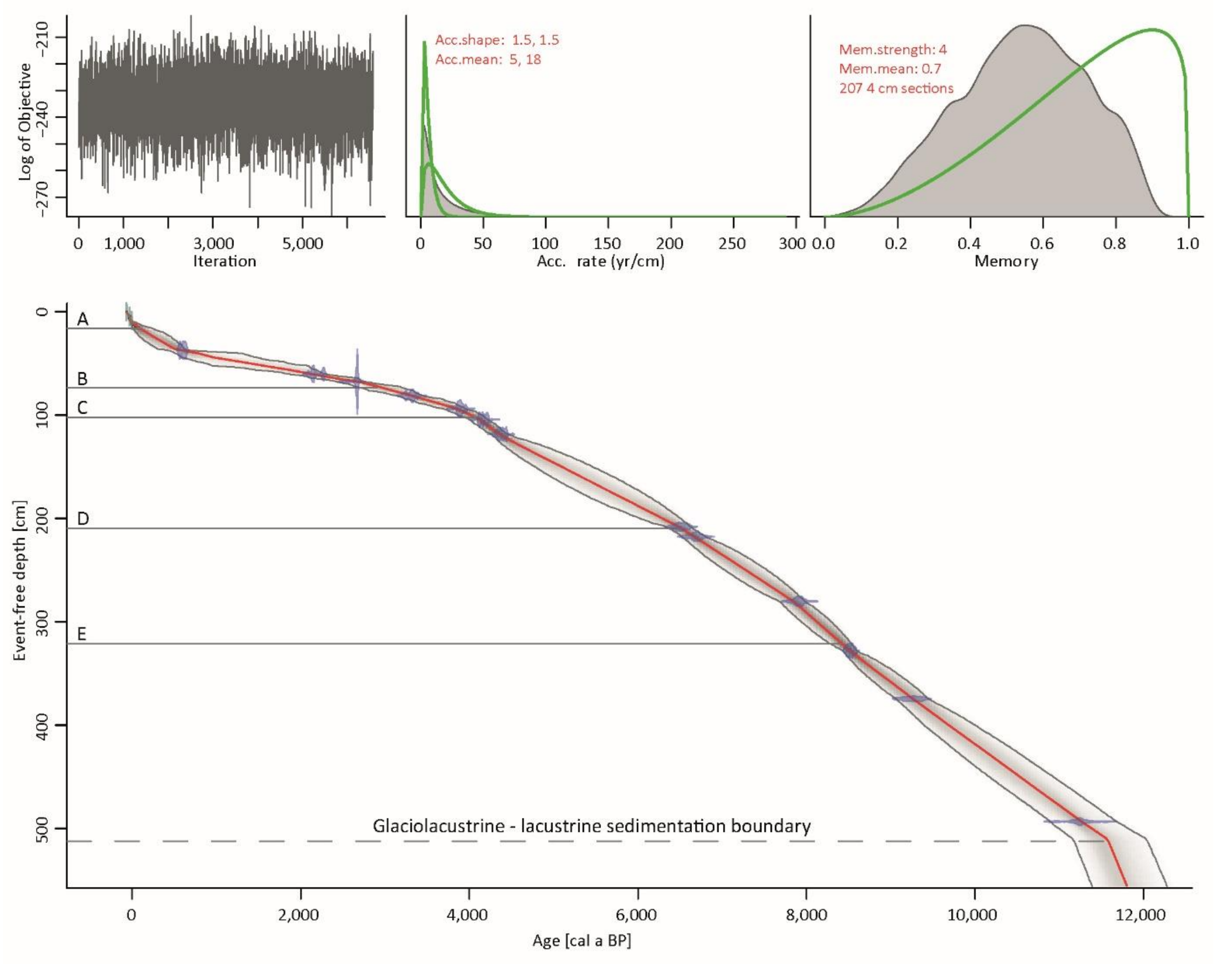

Supplementary Figure 12: Earthquake-induced turbidites in Plansee

A complete documentation of all amalgamated turbidites with core image (left) and CT data (right) in the Holocene record of Plansee interpreted as earthquake-induced. An amalgamated turbidite consists of at least two event beds, which share a single clay cap on top. Each individual event bed within the amalgamated turbidite usually consists of a homogeneous fine-grained silt package on top of a thin normal graded coarse (sandy-silt) base and fundamentally differs from overall < 5cm thick normal graded event deposits related to flood- and debris flow activity. Postseismic landscape response is interpreted based on the enhanced occurrence of flood- and debris flow deposits in combination with the change in sediment colour in the aftermath of the amalgamated turbidite compared to the overall lacustrine sedimentation. The size and number of flood- and debris flow deposits (green) decreases with time and sedimentation transits back to finely laminated lacustrine clayey silts. Postseismic landscape response can be explained by earthquake-triggered onshore mass-wasting leading to an enhanced availability of loose sediment in the catchment, which gets episodically transported into the lake during high river discharge events[22,27]. Location in core of each amalgamated turbidite is shown in Supplementary Figure 10. Event ages are shown in Supplementary Figure 11 and Supplementary Table 2.

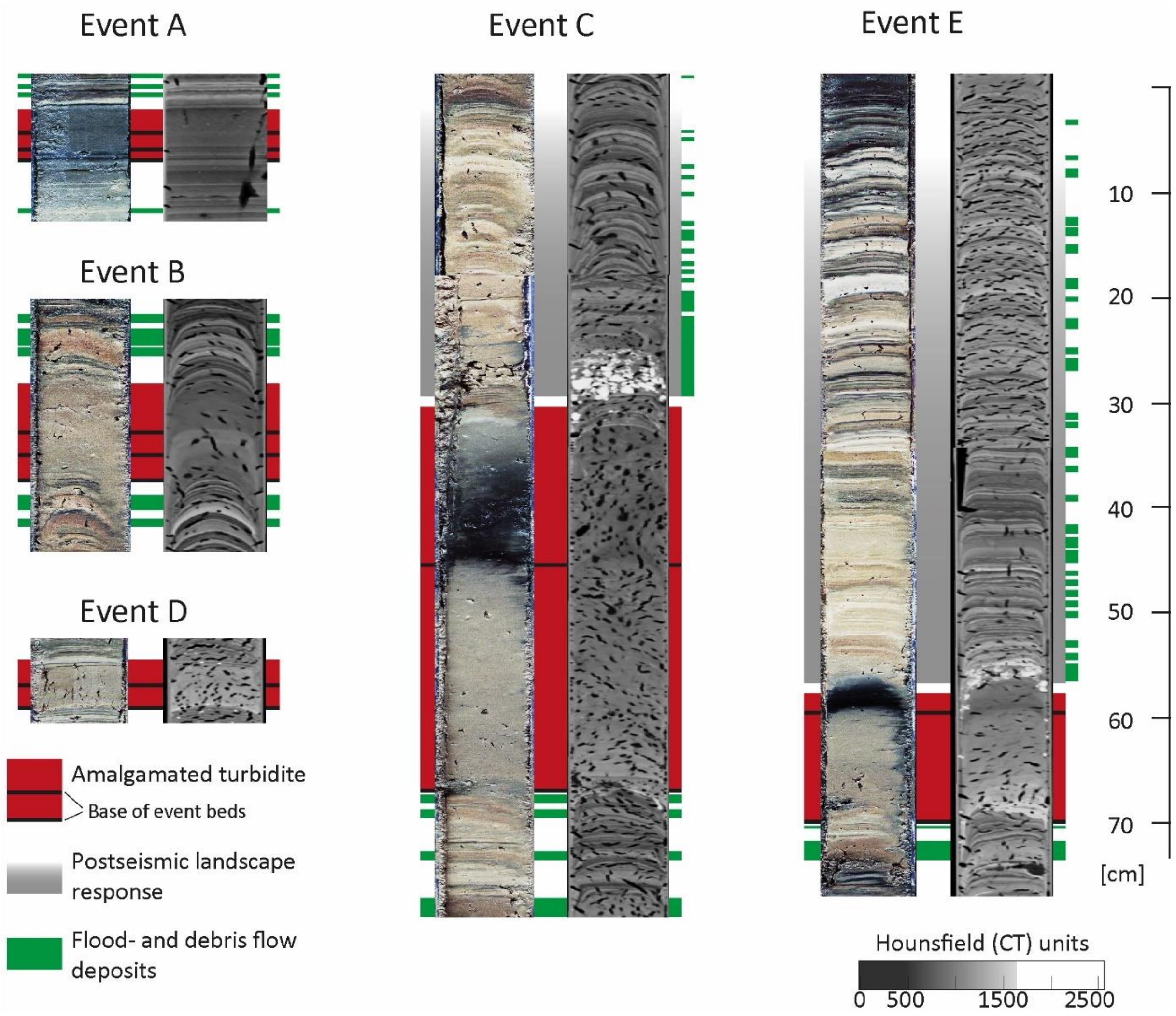

Supplementary Table 2 Plansee earthquake events

Earthquake ID	Event horizon	Core depth [cm]	Event corrected depth [cm]	Modelled event ages [cal a BP]				Turbidite thickness [cm]	Postseismic landscape response	MTD volume [m³]	Qualitative earthquake intensity
				mean	from	to	%				
EQ-A	A	21	16	CE 1930 Namlos earthquake			-	8	-	21,000	intermediate
EQ-B	B	96.5	73	2948	2691	3196	95	9	-	8,000	intermediate
EQ-C	C	142.5	102	4076	3961	4187	95	37	yes	100,000	high
EQ-D	D	310.5	209	6515	6372	6649	95	4	-	40,000	low
EQ-E	E	448.5	320.5	8422	8283	8543	95	16.5	yes	17,000	high

Supplementary Figure 13: Sedimentary imprint of the CE 1930 Namlos earthquake in Plansee

Activity measurements of natural (^{210}Pb) and artificial radionuclides (^{137}Cs) of the youngest sediments of Plansee and a constant flux constant sedimentation rate (CFCS) age-depth model. An amalgamated turbidite occurs at sediment depth 23 to 28 cm, consisting of three homogeneous fine-grained event beds on top of a thin normal graded coarse (sandy-silt) base and a shared fine-silt top (Supplementary Figure 12). These sedimentological observations indicate multiple, coeval mass wasting [28] and fundamentally differentiate the amalgamated turbidite from <5 cm thick, normal-graded event deposits related to flood- or debris flow activity [29]. The amalgamated turbidite links to multiple MTDs corresponding to event horizon A in the reflection seismic data (see Manuscript Figure 3a; Supplementary Figure 9). We measured activities of xs^{210}Pb and ^{137}Cs on the uppermost 50 cm of lacustrine clayey silts, while avoiding obvious event deposits (flood deposits and the amalgamated turbidite). xs^{210}Pb activities exponentially decay from 0 to 15 cm event-free sediment depth (middle panel). ^{137}Cs activities show two peaks at 5 cm and 8 cm representing the CE 1986 Chernobyl accident and the peak of atomic bomb tests in CE 1963, respectively. We applied a CFCS age-depth model on the ^{210}Pb activities on the upper 15 cm event-free sediment depth using SERAC[30] (right panel) resulting in a mean sedimentation rate of 2.25 mm/a (r^2 = 0.8875). The CFCS-model slightly underestimates the true age according to the position of the anthropogenic ^{137}Cs-peaks. Extrapolation of the xs^{210}Pb-model to the event-free sediment depth of the amalgamated turbidite at

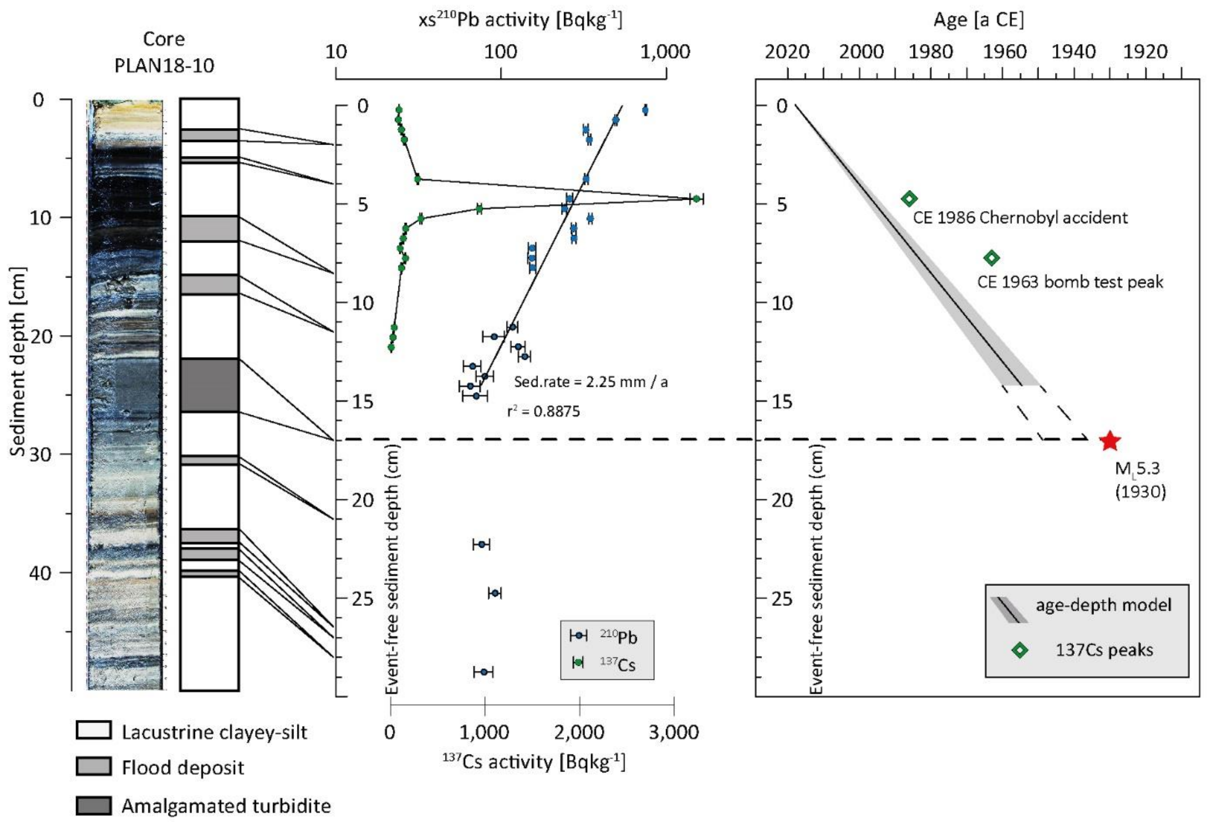

17 cm results in CE 1948 – 1936. Considering the offset to the ^{137}Cs-peaks and the simplicity of the model together with sedimentological constraints, we interpret the amalgamated turbidite to correspond with the M_L 5.3 earthquake in CE 1930.

Supplementary Table 3: Rockslide compilation

Rockslide	Age [cal a BP]	Error [a]	Age dating technique	Volume [x10^6 m^3]	Distance rockslide to Piburgersee [km]	Distance rockslide to Plansee [km]	Reference
Tschirgant	3000	62	^{14}C, U/Th; minimum and maximum ages	250	8	25	[31]
Haiming	3065	145	^{14}C; minimum age	60	8	23	[32]
Tumpen 1	3640	200	^{14}C; minimum age	-	2	33	[10]
Stöttlbach	3800	660	^{14}C; maximum age	30	16	19	[11]
Tumpen 2	3900	300	^{14}C; extrapolated age	35	3	34	[10]
Fernpass	4150	100	U/Th; maximum age	1,000	20	11	[33]
Eibsee	4025	55	^{14}C; event age	200	14	27	[34]
Habichen	4428	90	^{14}C; event age	-	1	32	this study
Köfels 2	7600	950	^{14}C	not distinguished from main event	9	40	[35]
Köfels	9512.5	14.5	Dendrochronology and ^{14}C wiggle matching; event age	3,280	9	40	[36]
Ehrwald	< 11700	-	No radiometric dating, geological observations	10-40	24	12	[11]

Supplementary Figure 14: Age overlap statistics on earthquake-induced event deposits in Piburgersee and Plansee at ~3.0 and ~4.1 ka BP

Overlap statistics of the Bayesian-modelled age probability density functions (PDFs) for the earthquake-induced event deposits in Piburgersee and Plansee at ~3.0 and ~4.1 ka BP (a) computed with the R-software package 'overlapping'[37]. Single-earthquake scenarios at ~3.0 and ~4.1 ka BP have an age overlap of 55% and 45%, respectively. Application of overlap statistics requires several assumptions regarding distribution symmetry or parametric family[37]. In our case, the position and shape of an event age PDF is strongly dependent on multiple parameters such as the age range of the nearest ^{14}C dates, the distance (i.e. core depth) between these radiocarbon samples and the event, and the age-modelling parameters itself e.g. number of iterations, memory strength and priors on sedimentation rate variability. Moreover, the width of the PDFs (age precision) can have an effect on the overlap statistics and may explain the lower overlap value for the 4.1 ka event, for which PDFs have important differences in width. Therefore, these overlap statistics only form a first-order

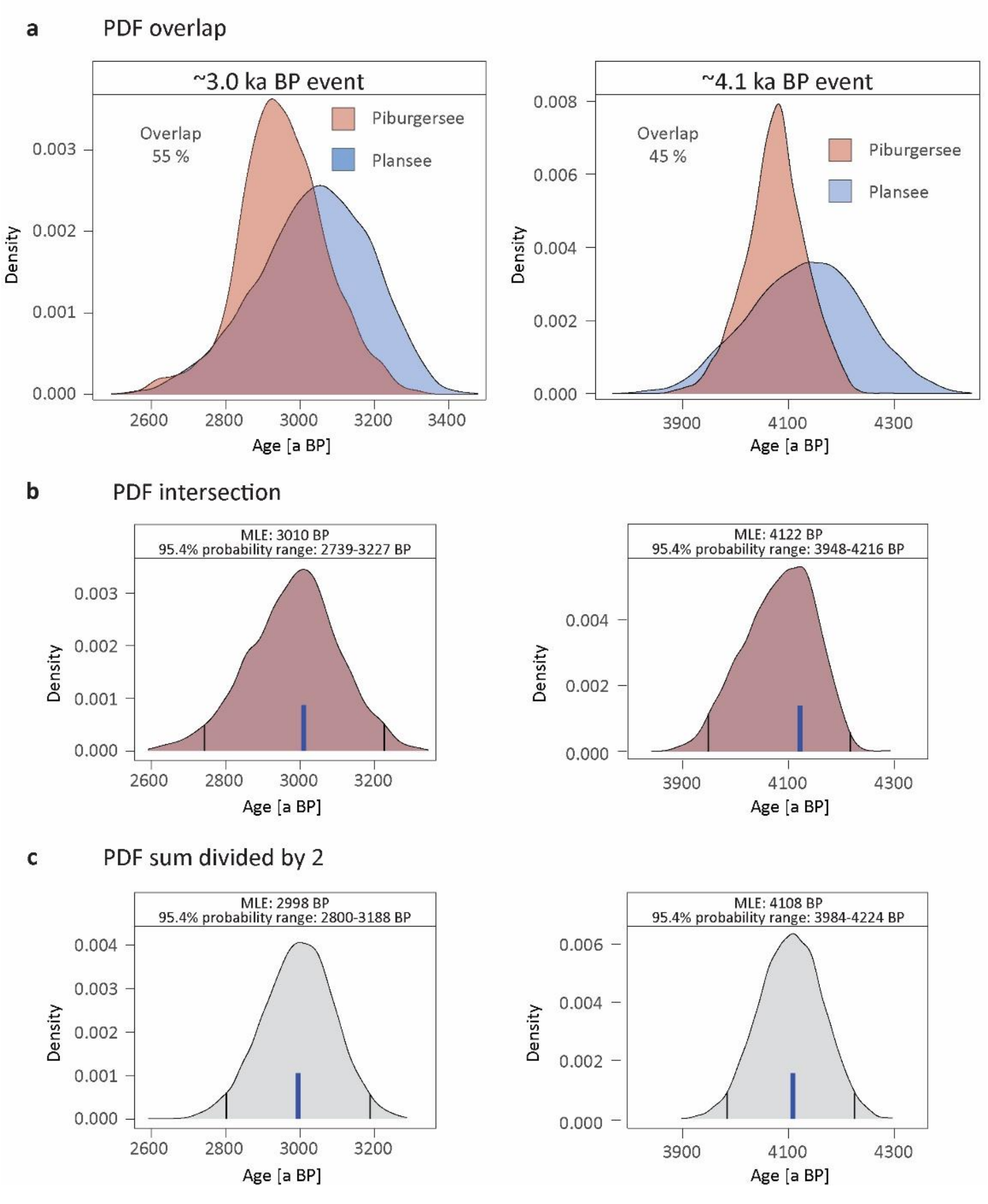

evaluation of potential synchronicity of ^{14}C-derived event layer ages. Maximum likelihood estimations (MLE) and 95.4% probability ranges are shown for the intersection of PDFs (b) and, by taking the sum of the PDFs divided by half (c). Both calculations result in very similar results, MLE 3010 BP, range 2739-3227 a BP compared to MLE 2998 a BP, range 2800-3188 a BP and MLE 4122 a BP, range 3948-4216 a BP compared to MLE 4108 a BP, range 3984-4224 a BP (b,c).

Supplementary Discussion 2

Hydro-climatic changes are among the most important preparation and trigger factors of rockslides[38] acting on long (total precipitation and frost weathering) and short time scales (extreme precipitation events). Frost weathering (i.e. ice segregation and volumetric expansion) drive fracture propagation in a rock mass and, along with permafrost degradation, can cause progressive weakening of a rock slope[39]. Extended periods of precipitation or extreme precipitation events lead to increased water tables and thus increased pore water pressures in the rock mass causing long-term hydromechanical fatigue[40]. Moreover, such reduced effective stress can also cause the final failure of a rock slope along prepared, potential shear surfaces.

To provide a conclusive assessment on the main trigger mechanism for the rockslides Fernpass and Eibsee at ~4.1 and Tschirgant and Haiming at ~3.0 ka BP (Manuscript Figures 1 and 4), the potential role of hydro-climate in triggering these rockslides must be evaluated. Therefore, we compiled Alpine-wide hydro-climatic proxy data and compared them to the rockslide ages and our paleoseismic data (Supplementary Figure 15). Precipitation in the Alps is concentrated on the southern or the northern rims of the Alpine chain depending on the source regions of the cyclones and their track[41]. Thus, the data from the northern Central Alps (c), the northern Eastern Alps (e, f, h) are more relevant for the study area, but data of the southern Central Alps (d) and Western Alps (g) are included to provide an Alpine-wide overview on the Holocene hydro-climate.

In general, hydro-climatic records of the Holocene indicate several periods of high flood occurrence for both the N- and S central Alps[42] (blue bars), during which we assume a higher importance of hydromechanical fatigue as preparatory factor for rockslides than during drier periods. However, seven out of ten large rockslides occurred in the period 4.4-3.0 ka BP which does not coincide with an inferred Alpine-wide nor with a regional wet period in the Eastern Alps. This mismatch is most striking for the Little Ice Age (CE 1350-1850) for which lacustrine records show one of the highest flood activities during the Holocene[42], but no rockslides are documented in the study area at this time.

A shift towards wetter climate was documented at 4.2 ka BP in the Southern and Western Alps (d, g). Accordingly, studies in the southern Western Alps attributed a series of rockslides to be hydro-climatically triggered during this '4.2 ka hydrological event'[43]. However, there is no evidence for an extraordinary wet period or more frequent extreme precipitation in the N- and E-Alps at this time (c, e, f, h). Therefore, a wet period or more frequent extreme precipitation can be reasonably excluded as main trigger for the ~4.1 ka BP rockslides (Fernpass and Eibsee). For the ~3.0 ka BP rockslides (Tschirgant and Haiming), proxy data are contradictory: the northern Eastern Alps show a relatively high flood activity (e) together with relatively enhanced winter precipitation (h), whereas the flood

frequency was low in the northern Central Alps (c), and both lacustrine records of our study suggest drier climatic conditions between ~3.8 and ~2ka BP given a substantial decrease in background (event-free) sedimentation rates (from 0.045 to 0.020 cm/a in Plansee and from 0.025 to 0.014 cm/a in Piburgersee; Supplementary Figure 3, 11). While neglecting the potential complexity caused by the different nature of these records (winter precipitation, spring-summer floods and lacustrine sedimentation rate), neither of the records indicating a wet period at 3.0 ka BP (d, h) shows an elevated proxy amplitude at that time in comparison to other periods in these records. Additionally, these records show no significant trend change at 3.0 ka BP (d, h), as is the case for the 4.2 ka BP hydrological event in the Southern and Western Alps (f, g) that forms the scientific base for attributing rockslides to hydro-climatic change in the SW Alps[43]. Thus, there is also currently no proxy evidence supporting a significant hydro-climatic influence for triggering the 3.0 ka BP rockslides.

The upper source areas of the herein investigated rockslides are well above 2,000 m asl and are snow- or ice covered in the winter months even during nowadays relatively warm temperatures (h). Thus, frost weathering may have gradually affected these rock slopes during similar or lower temperatures in the Holocene. However, as these weakening processes are rather surficial in a rock slope[44] and the rockslides in our study area are deep-seated, frost weathering cannot solely cause a spatiotemporal clustering of deep-seated rockslides.

It can be expected that wet periods, extreme precipitation events and frost weathering were relevant in long-term rock mass weakening and failure preparation of the investigated rockslides. However, based on the above considerations there is no conclusive evidence for inferring a causal relationship between wet periods or extreme precipitation and rockslide activity in the Eastern Alps. In contrast, the striking spatiotemporal coincidence of rockslides and paleo-earthquakes in the study area at 4.1 and 3.0 ka BP (a, b; Manuscript Figure 1; Supplementary Figure 14) attest that these rockslides were triggered by severe seismic shaking.

Supplementary Figure 15: Comparison of the rockslide events with our paleoseismic records and Alpine-wide hydro-climatic proxy data

Ages of rockslides (a; see Supplementary Table 3) and paleoseismic data (b; see Supplementary Tables 1,2) are compared to Alpine-wide hydro-climatic proxy data (numbers in brackets represent mean distance of catchment area to study area). We displayed two Holocene flood record compilations for the Central Alps[42] as a proxy for heavy precipitation events and separate N-Central Alps (c) from S-Central Alps (d) in order to cover precipitation patterns north and south of the Alpine divide. In addition, a river flood record of Ammersee (e)[45] and the Piburgersee flood record (f) are shown, representing flood activity in the northern Eastern Alps, respectively. To capture total precipitation changes in the Holocene, a record of lake level changes from lake Cerin in the Western Alps (g)[46] and a speleothem record from the central Eastern Alps (h)[47] are shown. The latter is interpreted to represent cold conditions and high winter precipitation reflected by low $\delta^{18}O$ values and vice versa. Orange bars indicate the proposed timing of synchronous rockslides at ~4.1 and ~3.0 ka BP, light orange bars highlight the period of enhanced rockslide activity at 4.4-3.0 ka BP and light blue bars indicate high flood occurences in the northern and southern Central Alps[42]. The inset in the lower right shows the spatial distribution of the proxy data (a-h).

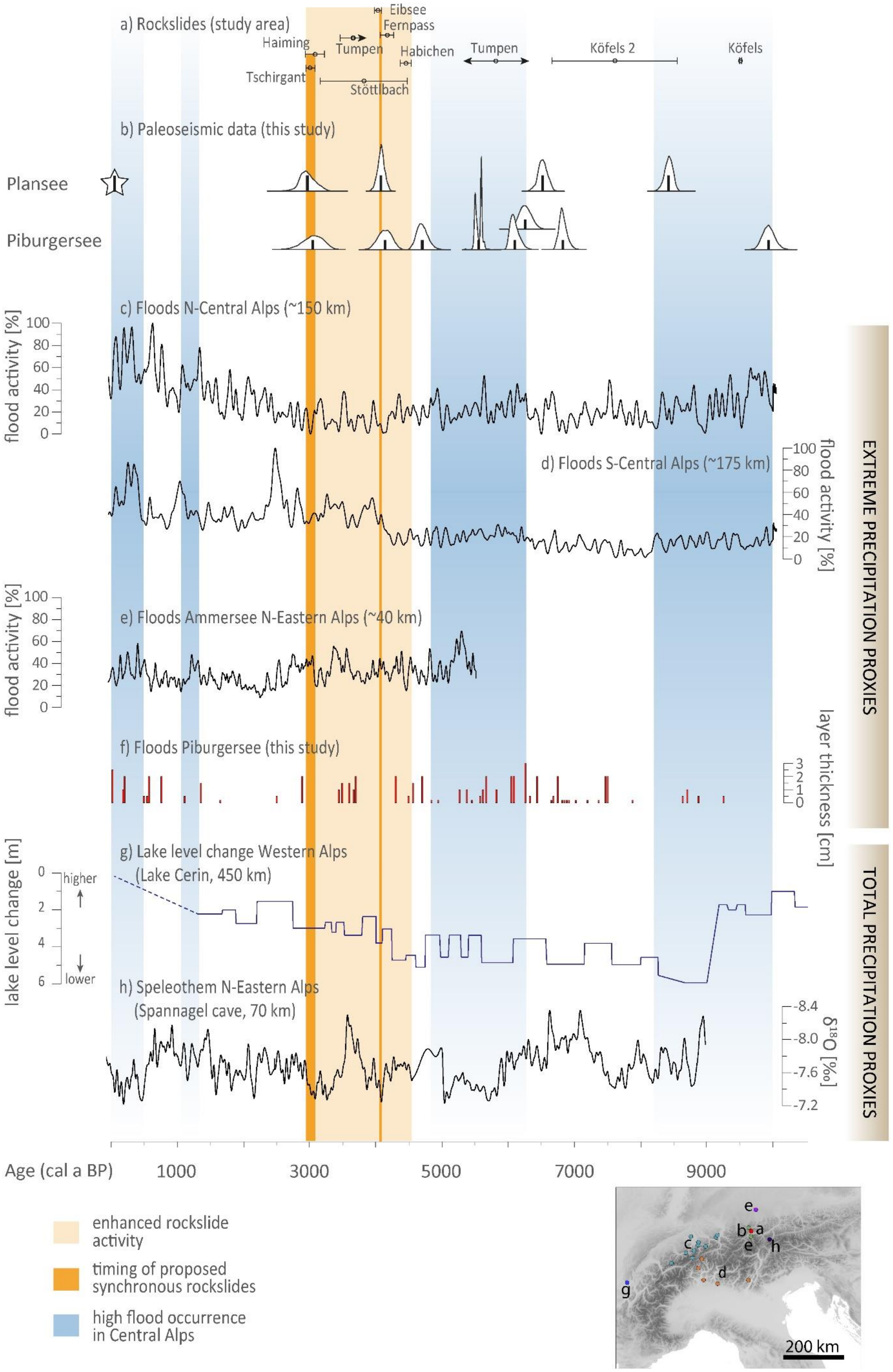
a) Rockslides (study area)
Eibsee
Fernpass
Haiming
Tumpen
Habichen
Tumpen
Köfels 2
Köfels
Tschirgant
Stöttlbach
b) Paleoseismic data (this study)
Plansee
Piburgersee
c) Floods N-Central Alps (~150 km)
flood activity [%]
d) Floods S-Central Alps (~175 km)
e) Floods Ammersee N-Eastern Alps (~40 km)
f) Floods Piburgersee (this study)
layer thickness [cm]
g) Lake level change Western Alps (Lake Cerin, 450 km)
lake level change [m]
higher
lower
h) Speleothem N-Eastern Alps (Spannagel cave, 70 km)
δ18O [‰]
EXTREME PRECIPITATION PROXIES
TOTAL PRECIPITATION PROXIES
Age (cal a BP)
1000
3000
5000
7000
9000
enhanced rockslide activity
timing of proposed synchronous rockslides
high flood occurrence in Central Alps
200 km

426

Supplementary Discussion 3

Evaluation of synchronicity of paleoseismic events obtained in multiple paleoseismic archives is an important challenge in lacustrine paleoseismology and has significant implications on the estimation of possible magnitudes and epicentre locations for the respective single earthquake or multiple earthquakes scenario[19]. In our study, a multiple-earthquakes scenario for the ~4.1 and ~3.0 ka BP periods would lower the inferred minimum magnitude M_L 5.5 (Supplementary Text 2) to the documented M_L 5.3 of the CE 1930 Namlos earthquake (see also Supplementary Figure 6). This would have little influence for our interpretation of seismic triggering of the large rockslides at ~4.1 and ~3.0 ka BP, as in a multiple-earthquake scenario, the rockslides in the North would have been triggered by severe seismic shaking in the North at ~4.1 ka BP followed by a subsequent smaller earthquake in the South (but above intensity VI at Piburgersee), and vice versa for the ~3.0 ka BP event.

Supplementary Method 1: Minimum magnitude calculation

The minimum magnitude for a single-earthquake scenario reaching seismic intensities > VI at both lakes (Piburgersee and Plansee) was calculated using the latest Austrian intensity prediction equation (IPE; equation (1)[48]), where I_{local} is local intensity, M_W moment magnitude, h depth in km, R distance in km and k_0 (2.56), k_1 (1.32), k_2 (0.94) and c_0 (-1.05) are empirical constants. The resulting moment magnitudes M_W are converted to local magnitudes M_L using the latest empirical Austrian relation (equation (2)[49]. In order to obtain a minimum estimate for a single earthquake scenario, we assumed the epicentre location exactly in the middle between the two lakes (30 km lake-to-lake distance), which lead to a minimum M_L 5.5. This hypothetic epicentre location is justified as it lies within the area of highest present-day seismicity[1] (Supplementary Figure 1). Relocation of this hypothetic epicentre in any direction would result in a larger magnitude for the paleo-earthquake event.

$$(1)\ I_{local} = k_0 + k_1 M_W + k_2 \ln(h) + c_0 \ln(R/h)$$

$$(2)\ M_W = M_L - 0.3 \qquad |\ \mathrm{M_L} \geq 4$$

Supplementary Table 4 Radiocarbon and radionuclide samples of Piburgersee

Core ID	Sample no.	Core depth (cm)	Radiocarbon age (a BP ± 1σ)	95% calibrated age range (cal a BP)	Material
PIBU18-01	-	10	-	83-73	210Pb/137Cs extrapolated age[4]
PIBU18-01	ETH-94775	54.5	882 ± 21	903-733	fir needles
PIBU18-01	ETH-94776	82.5	2493 ± 22	2720-2489	leaf and fir needles
PIBU18-01*	ETH-92029	81.5	6457 ± 23	7427-7323	fir needles
PIBU18-L1A-3-4.5	ETH-94777	353.5	3241 ± 22	3557-3395	fir needles, fir cone remains, betula fruit
PIBU18-L1A_3-4.5	ETH-92030	370.5	3515 ± 23	3860-3705	leaf and fir needles
PIBU18-L1A-3-4.5	ETH-94778	383.5	3983 ± 22	4519-4416	needles and Carpinus Betulus fruit
PIBU18-L1A-4.5-6	ETH-92031	450	4060 ± 22	4784-4440	fir needles
PIBU18-L1B-4-5.5*	ETH-94779	456	4519 ± 23	5302-5053	fir needles, betula fruit, tree leaf stalk
PIBU18-L1B-4-5.5	ETH-94780	493.5	4828 ± 23	5605-5482	fir needles, tree leaf stalk
PIBU18-L1B-4-5.5	ETH-94781	511	5244 ± 23	6174-5926	fir needles
PIBU18-L1A-4.5-6*	ETH-94782	528	6005 ± 23	6925-6757	leaf remains, needles, betula fruit
PIBU18-L1A-4.5-6	ETH-94783	551.5	5933 ± 23	6830 – 6675	needles, twig remains
PIBU18-L1B-5.5-7*	ETH-92032	571.5	2499 ± 21	2724-2490	birch fruits, leafs, fir needles
PIBU18-L1B-5.5-7	ETH-96886	638	8310 ± 24	9429-9260	leaf fragments, fir needles
PIBU18-L1A-6-7.5	ETH-94784	650	8762 ± 25	9899-9634	leaf, twig, betula fruit
PIBU18-L1A-6-7.5	ETH-96887	671	9299 ± 26	10575-10419	leaf stalk
PIBU18-L1A-6-7.5	ETH-92033	691	10019 ± 26	11701-11335	twig and leaf remains, fir needle, birch fruits
PIBU18-L1B-7-8.5	ETH-92034	743	11025 ± 27	13004-12784	fir needles and birch fruit

* Samples excluded for age-depth modelling

Supplementary Table 5 Radiocarbon and radionuclide samples of Plansee

Core ID	Sample no.	Core depth (cm)	Radiocarbon age (a BP ± 1σ)	95% calibrated age range (cal a BP)	Material
Plan18-10	-	4.5	-	-36	137Cs peak
Plan18-10	-	8.5	-	-13	137Cs peak
PLAN18-L1A-0-1.5	ETH-101431	52	595 ± 35	654-538	leaf and fir needle remains
PLAN18-L1A-0-1.5	ETH-101432	81.5	2154 ± 24	2181-2058	leaf and fir needles
PLAN18-10	ETH-94774	89	2591 ± 22	2756-2720	needle and fir cone piece
PLAN18-L1A-0-1.5	ETH-101433	116.5	3136 ± 43	3449-3241	fir needle remains
PLAN18-L1A-0-1.5	ETH-103730	129	3581 ± 26	3972-3831	fir needle
PLAN18-L1B-1-2.5	ETH-103731	183.5	3782 ± 24	4236-4089	fir cone scale, needle remains
PLAN18-L1A-1.5-3	ETH-101434	208	3915 ± 25	4423-4285	leaf and fir needles
PLAN18-L1B-2.5-4	ETH-101435	309	5718 ± 56	6855-6403	leaf and fir needles
PLAN18-L1A-3-4.5	ETH-101436	322.5	5869 ± 57	6800-6529	fir needle remains
PLAN18-L1A-3-4.5	ETH-101437	399	7084 ± 65	8021-7783	fir needle remains
PLAN18-L1A-4.5-6	ETH-103732	473	7739 ± 29	8589-8443	leaf and fir needle remains
PLAN18-L1B-4-5.5*	ETH-101438	436.5	2595 ± 24	2759-2720	fir needles
PLAN18-L1B-4-5.5*	ETH-101439	473.5	3934 ± 25	4440-4288	pair of fir needles
PLAN18-L1A-4.5-6	ETH-101440	524	8278 ± 75	9460-9077	fir needle remains
PLAN18-L1A-6-7.5	ETH-101441	660	9800 ± 87	11412-11068	betula fruit, fir needle remains

*Samples excluded for age-depth modelling

Supplementary Table 6: M_L 5.1 Nassereith earthquake in 1886 - intensity data points

ML 5.1 earthquake in Nasserreith at 1886-11-28 / 10:30 p.m.

location	latitude	longitude	local intensity (EMS-98)
Bichlbach	47.418	10.792	5.5
Flaurling	47.291	11.122	4.0
Fliess	47.122	10.629	4.0
Füssen	47.569	10.704	1.0
Garmisch Patenkirchen	47.492	11.086	1.0
Hall in Tirol	47.289	11.507	1.0
Imst	47.243	10.742	6.0
Innsbruck	47.269	11.397	3.5
Karrösten	47.225	10.768	5.5
Kematen	47.254	11.272	4.0
Kufstein	47.583	12.173	3.0
Meran	46.669	11.164	3.0
Mötz	47.283	10.957	5.0
Nassereith	47.317	10.840	7.5
Obsteig	47.298	10.928	4.0
Pfelders	46.796	11.091	4.0
Riffian	46.702	11.181	4.0
Roppen	47.218	10.814	5.0
Seefeld in Tirol	47.330	11.186	4.5
Starkenberg	47.262	10.754	6.0
Steinach am Brenner	47.093	11.466	4.0
Sterzing	46.899	11.428	3.5
Stockach	47.261	10.38	4.0
Tarrenz	47.264	10.763	6.0
Telfs	47.309	11.075	4.0
Wenns	47.171	10.734	4.0
Wolfegg	47.261	10.173	5.0

Supplementary References

1. Reiter, F. *et al.* Active Seismotectonic Deformation in Front of the Dolomites Indenter, Eastern Alps. *Tectonics* **37**, 4625–4654 (2018).

2. Ortner, H., Reiter, F. & Brandner, R. Kinematics of the Inntal shear zone-sub-Tauern ramp fault system and the interpretation of the TRANSALP seismic section, Eastern Alps, Austria. *Tectonophysics* **414**, 241–258 (2006).

3. Tollmann, A. *Der Bau der Nördlichen Kalkalpen. Orogene Stellung Regionale Tektonik*. (Franz Deuticke, 1976).

4. Thies, H. *et al.* Interactions of temperature and nutrient changes: Effects on phytoplankton in the Piburger See (Tyrol, Austria). *Freshw. Biol.* **57**, 2057–2075 (2012).

5. Elfenbein, C. Bericht 1996 über geologische Aufnahmen in den Nördlichen Kalkalpen auf Blatt 115 Reutte. *Jahrb. der Geol. Bundesanstalt* **140**, 310–311 (1997).

6. Dietrich, A. & Krautblatter, M. Evidence for enhanced debris-flow activity in the Northern Calcareous Alps since the 1980s (Plansee, Austria). *Geomorphology* **287**, 144–158 (2017).

7. Huang, J. J. *et al.* Disentangling natural and anthropogenic signals in lacustrine records: An example from the Ilan Plain, NE Taiwan. *Front. Earth Sci.* **4**, 1–12 (2016).

8. Weltje, G. J. & Tjallingii, R. Calibration of XRF core scanners for quantitative geochemical logging of sediment cores: Theory and application. *Earth Planet. Sci. Lett.* **274**, 423–438 (2008).

9. Blaauw, M. & Christen, J. A. Flexible paleoclimate age-depth models using an autoregressive gamma process. *Bayesian Anal.* **6**, 457–474 (2011).

10. Poscher, G. & Patzelt, G. Sink-hole Collapses in Soft Rocks. *Felsbau, Rock Soil Eng.* **18**, 36–40 (2000).

11. Prager, C., Zangerl, C., Patzelt, G. & Brandner, R. Age distribution of fossil landslides in the Tyrol (Austria) and its surrounding areas. *Nat. Hazards Earth Syst. Sci.* **8**, 377–407 (2008).

12. Wetzler, N., Marco, S. & Heifetz, E. Quantitative analysis of seismogenic shear-induced turbulence in lake sediments. *Geology* **38**, 303–306 (2010).

13. Moernaut, J. *et al.* The subaqueous landslide cycle in south-central Chilean lakes: The role of tephra, slope gradient and repeated seismic shaking. *Sediment. Geol.* **381**, 84–105 (2019).

14. Van Daele, M. *et al.* Multidirectional, synchronously-triggered seismo-turbidites and debrites

revealed by X-ray computed tomography (CT). *Sedimentology* **61**, 861–880 (2014).

15. Agnon, A., Migowski, C. & Marco, S. Intraclast Breccias in Laminated Sequences Reviewed: Recorders of Paleo-earthquakes. *Spec. Pap. 401 New Front. Dead Sea Paleoenviron. Res.* 195–214 (2006). doi:10.1130/2006.2401(13)

16. Hammerl, C. Historical earthquake research in Austria. *Geosci. Lett.* **4**, 99 (2017).

17. Kraus, E. Die Bewegung des Erdbebens am 8. Oktober 1930 im süddeutschen Bau. *Berichte des naturwissenschaftlichen Vereins für Schwaben und Neubg.* **50**, (1932).

18. Monecke, K. *et al.* Earthquake-induced deformation structures in lake deposits: A Late Pleistocene to Holocene paleoseismic record for Central Switzerland. *Eclogae Geol. Helv.* **99**, 343–362 (2006).

19. Kremer, K. *et al.* Lake-sediment based paleoseismology: Limitations and perspectives from the Swiss Alps. *Quat. Sci. Rev.* **168**, 1–18 (2017).

20. Van Daele, M. *et al.* A comparison of the sedimentary records of the 1960 and 2010 great Chilean earthquakes in 17 lakes: Implications for quantitative lacustrine palaeoseismology. *Sedimentology* **62**, 1466–1496 (2015).

21. Moernaut, J. Time-dependent recurrence of strong earthquake shaking near plate boundaries: A lake sediment perspective. *Earth-Science Rev.* **210**, 103344 (2020).

22. Avşar, U., Jónsson, S., Avşar, Ö. & Schmidt, S. Earthquake-induced soft-sediment deformations and seismically amplified erosion rates recorded in varved sediments of Köyceğiz Lake (SW Turkey). *J. Geophys. Res. Solid Earth* **121**, 4767–4779 (2016).

23. Schnellmann, M., Anselmetti, F. S., Giardini, D., Mckenzie, J. A. & Ward, S. N. Prehistoric earthquake history revealed by lacustrine slump deposits. *Geology* **30**, 1131–1134 (2002).

24. Strasser, M., Monecke, K., Schnellmann, M. & Anselmetti, F. S. Lake sediments as natural seismographs: A compiled record of Late Quaternary earthquakes in Central Switzerland and its implication for Alpine deformation. *Sedimentology* **60**, 319–341 (2013).

25. Girardclos, S. *et al.* The 1996 AD delta collapse and large turbidite in Lake Brienz. *Mar. Geol.* **241**, 137–154 (2007).

26. Praet, N. *et al.* Paleoseismic potential of sublacustrine landslide records in a high-seismicity setting (south-central Alaska). *Mar. Geol.* **384**, 103–119 (2017).

27. Howarth, J. D., Fitzsimons, S. J., Norris, R. J., Langridge, R. & Vandergoes, M. J. A 2000 yr

rupture history for the Alpine fault derived from Lake Ellery, South Island, New Zealand. *Bull. Geol. Soc. Am.* **128**, 627–643 (2016).

28. Van Daele, M. *et al.* A revised classification and terminology for stacked and amalgamated turbidites in environments dominated by (hemi)pelagic sedimentation. *Sediment. Geol.* **357**, 72–82 (2017).

29. Wilhelm, B. *et al.* Does global warming favour the occurrence of extreme floods in European Alps? First evidences from a NW Alps proglacial lake sediment record. *Clim. Change* **113**, 563–581 (2012).

30. Bruel, R. & Sabatier, P. serac: A R package for ShortlivEd RAdionuclide chronology of recent sediment cores. *J. Environ. Radioact.* **225**, 106449 (2020).

31. Ostermann, M., Ivy-Ochs, S., Sanders, D. & Prager, C. Multi-method (14 C, 36 Cl, 234 U/ 230 Th) age bracketing of the Tschirgant rock avalanche (Eastern Alps): Implications for absolute dating of catastrophic mass-wasting. *Earth Surf. Process. Landforms* **42**, 1110–1118 (2017).

32. Patzelt, G. Die Bergstürze vom Tschirgant und von Haiming, Oberinntal, Tirol Begleitworte zur Kartenbeilage. *Jahrb. der Geol. Bundesanstalt* **1**–**4**, 13–24 (2012).

33. Prager, C., Ivy-Ochs, S., Ostermann, M., Synal, H.-A. & Patzelt, G. Geology and radiometric 14C-, 36Cl- and Th-/U-dating of the Fernpass rockslide (Tyrol, Austria). *Geomorphology* **103**, 93–103 (2009).

34. Knapp, S., Anselmetti, F. S., Lempe, B. & Krautblatter, M. Impact of an 0.2 km3 Rock Avalanche on Lake Eibsee (Bavarian Alps, Germany) – Part II: Catchment Response to Consecutive Debris Avalanche and Debris Flow. *Earth Surf. Process. Landforms* (2020). doi:10.1002/esp.5025

35. Ivy-Ochs, S. *et al.* The age of the Köfels event-relative, 14C and cosmogenic isotope dating of an early Holocene landslide in the Central Alps (Tyrol, Austria). *Zeitschrift für Gletscherkd. und Glazialgeol.* **1**, 57–68 (1998).

36. Nicolussi, K., Spötl, C., Thurner, A. & Reimer, P. J. Precise radiocarbon dating of the giant Köfels landslide (Eastern Alps, Austria). *Geomorphology* **243**, 87–91 (2015).

37. Pastore, M. & Calcagnì, A. Measuring distribution similarities between samples: A distribution-free overlapping index. *Front. Psychol.* **10**, 1–8 (2019).

38. Pánek, T. Landslides and Quaternary climate changes—The state of the art. *Earth-Science Rev.* **196**, 102871 (2019).

39. Draebing, D. & Krautblatter, M. The Efficacy of Frost Weathering Processes in Alpine Rockwalls. *Geophys. Res. Lett.* **46**, 6516–6524 (2019).

40. Preisig, G., Eberhardt, E., Smithyman, M., Preh, A. & Bonzanigo, L. Hydromechanical rock mass fatigue in deep-seated landslides accompanying seasonal variations in pore pressures. *Rock Mech. Rock Eng.* **49**, 2333–2351 (2016).

41. Isotta, F. A. *et al.* The climate of daily precipitation in the Alps: Development and analysis of a high-resolution grid dataset from pan-Alpine rain-gauge data. *Int. J. Climatol.* **34**, 1657–1675 (2014).

42. Wirth, S. B., Glur, L., Gilli, A. & Anselmetti, F. S. Holocene flood frequency across the Central Alps - solar forcing and evidence for variations in North Atlantic atmospheric circulation. *Quat. Sci. Rev.* **80**, 112–128 (2013).

43. Zerathe, S., Lebourg, T., Braucher, R. & Bourlès, D. Mid-Holocene cluster of large-scale landslides revealed in the Southwestern Alps by 36Cl dating. Insight on an Alpine-scale landslide activity. *Quat. Sci. Rev.* **90**, 106–127 (2014).

44. Matsuoka, N. & Murton, J. Frost weathering: recent advances and future directions. *Permafr. Periglac. Process.* **19**, 195–210 (2008).

45. Czymzik, M. *et al.* Orbital and solar forcing of shifts in Mid- to Late Holocene flood intensity from varved sediments of pre-alpine Lake Ammersee (southern Germany). *Quat. Sci. Rev.* **61**, 96–110 (2013).

46. Magny, M., Bossuet, G., Ruffaldi, P., Leroux, A. & Mouthon, J. Orbital imprint on Holocene palaeohydrological variations in west-central Europe as reflected by lake-level changes at Cerin (Jura Mountains, eastern France). *J. Quat. Sci.* **26**, 171–177 (2011).

47. Mangini, A. *et al.* Persistent influence of the North Atlantic hydrography on central European winter temperature during the last 9000 years. *Geophys. Res. Lett.* **34**, PA2003 (2007).

48. Papí Isaba, M. del P. *et al.* Intensity Prediction Equation for Austria: Applications and analysis. *EGU Gen. Assem. 2020, Online, 4–8 May 2020* (2020). doi:https://doi.org/10.5194/egusphere-egu2020-7683

49. Weginger, S., Papí Isaba, M. del P., Jia, Y. & Lenhardt, W. Seismic hazard map of Austria. *EGU Gen. Assem. 2020, Online, 4–8 May 2020* (2020). doi:https://doi.org/10.5194/egusphere-egu2020-4820
