# Supplementary material for: Seismic control of large prehistoric rockslides in the Eastern Alps
Source: Nat Commun. 2021 Feb 16;12:1059. doi: 10.1038/s41467-021-21327-9 (PMC7886888; doi:10.1038/s41467-021-21327-9)
Supplement: Supplementary file 3 — Description of Additional Supplementary Files [file 41467_2021_21327_MOESM3_ESM.pdf]

Description of Additional Supplementary Files

File name: Supplementary Movie 1
Description: 3D visualization of soft sediment deformation structures (SSDS) in Piburgersee using X-ray computer tomographic data

File name: Supplementary Movie 2
Description: 3D visualization of an earthquake-induced amalgamated turbidite and its overlying postseismic landscape response in Plansee (Event C) using X-ray computer tomographic data
